# Advanced Analytical Framework for Pyrolysis Product Characterization and Emission Profiling in Mixed Plastic Waste: Implications for Recycling Strategy

**DOI:** 10.3390/polym18111381

**Published:** 2026-06-02

**Authors:** Aiping Chen, Saumitra Saxena, Vasileios G. Samaras, Bassam Dally

**Affiliations:** 1 Clean Energy Research Platform, Physical Science and Engineering Division (PSE), King Abdullah University of Science and Technology, Thuwal 23955-6900, Saudi Arabia; aiping@mail.ust.edu.cn (A.C.); bassam.dally@kaust.edu.sa (B.D.); 2 Analytical Chemistry Core Lab, King Abdullah University of Science and Technology, Thuwal 23955-6900, Saudi Arabia; vasileios.samaras@kaust.edu.sa

**Keywords:** mixed plastic waste, plastic pyrolysis oil, chemical recycling, comprehensive two-dimensional gas chromatography, non-intentionally added substances (NIASs), food-contact compliance, extractables and leachables

## Abstract

Chemical recycling of mixed plastic waste can recover hydrocarbon products, but additive-derived non-intentionally added substances (NIASs) and other volatile or extractable residues may affect product quality and safety. In this study, six polyolefin-rich waste streams (P1–P6) were analyzed by analytical pyrolysis coupled with comprehensive two-dimensional gas chromatography-time-of-flight mass spectrometry (Py–GC×GC–TOF–MS), while three additional consumer-grade plastics (P7–P9) were examined by headspace/solvent-extraction GC–MS and aqueous migration testing to profile volatile organic compounds (VOCs), semi-volatile organic compounds (SVOCs), and water migrants. Under rapid pyrolysis at 650 °C, the condensable products were dominated by C5–C30 aliphatic hydrocarbons. Polyethylene (PE)-rich feeds produced mainly n-paraffins and α-olefins, whereas polypropylene (PP)-rich feeds produced more branched olefins and modest mono-aromatics. Oxygenated compounds were negligible in non-oxidized feeds, but persisted at low levels in weathered high-density polyethylene (HDPE), consistent with pre-existing oxidation. Antioxidant-derived NIASs, including 2,4-di-tert-butylphenol and an Irganox 1010-related spiro-dione, were detected at trace to low area-fraction levels. VOC/SVOC and migration analyses revealed mainly low-intensity hydrocarbons, esters, antioxidant-related degradation products, caprolactam, and selected plasticizer-related compounds. These results show that relatively clean polyolefin streams can yield hydrocarbon-rich pyrolysates, but oxidized PE and additive-derived NIASs remain important quality-control targets. The GC-based methods used here characterize the volatile, condensable, and readily extractable fraction and do not represent the total contaminant load of the source waste.

## 1. Introduction

The global shift toward a circular plastic economy [1] has intensified focus on chemical recycling technologies like pyrolysis, which can convert mixed or contaminated plastic waste into reusable hydrocarbon feedstocks [2]. However, realizing this potential requires a deep understanding of how legacy contaminants and additives present in waste streams behave during thermal conversion [2,3,4,5,6,7,8,9,10,11]. A decisive policy driver behind this work is the UN-led Global Plastics Treaty now under negotiation (INC-5, Busan, 2025) [12], which aims to deliver a legally binding agreement that tackles plastic pollution across the entire life-cycle, including strict control of hazardous additives and full chemical transparency for recycled outputs [13,14,15,16].

The present manuscript addresses two related but distinct questions. First, how do differences in polyolefin-rich waste feeds translate into pyrolysis oil fingerprints, additive-derived NIASs, and low-level oxygenated species? Second, what volatile, semi-volatile, and water migrating compounds remain associated with selected recycled plastic articles relevant to downstream use? Although the same sample identifiers were used in a previously published study by our group [17], the present work is self-contained: the key feed descriptors required for interpretation are summarized again in Table 1.

While both studies share the same sample identifiers, the analytical scope differs substantially: our previously published study [17] focuses on feedstock characterization by FTIR, TGA/DTG, DSC, ICP–OES, and XRF, whereas the present work applies Py–GC×GC–TOF–MS, headspace GC–MS, solvent-extraction GC–MS, and water migration testing to the pyrolysis oils and recycled articles derived from those feeds. No raw data are duplicated between the two manuscripts, and each paper is designed to be independently readable.

The growing interest in chemical recycling of polyolefin waste has been driven by increasingly stringent regulatory frameworks and the recognition that mechanical recycling alone cannot close the plastics loop. The European Packaging and Packaging Waste Regulation (EU/2025/40), which enters force in August 2026, mandates minimum recycled content thresholds for food-contact plastics and requires systematic minimization of substances of concern (SoCs) in packaging, including per- and polyfluoroalkyl substances at concentrations above 25 ppb (individual) or 250 ppb (sum) [18]. In parallel, the European Food Safety Authority (EFSA) updated its scientific guidance for recycled plastic safety assessment in 2024, requiring that contaminant exposure from recycled food-contact materials remain below 0.0025 μg/kg body weight per day, although this guidance currently applies primarily to mechanical PET recycling and offers limited direction for polyolefin pyrolysis pathways [19]. The US FDA similarly requires surrogate-contaminant testing and migration studies demonstrating removal to safe levels (≤0.5 ppb for specific contaminants), yet chemical recycling-specific criteria for pyrolysis oils remain minimal [1]. This regulatory asymmetry—where mechanical recycling routes are increasingly codified while chemical recycling via pyrolysis operates in a less defined regulatory space—underscores the urgent need for robust analytical characterization of pyrolysis products.

Pyrolysis oils derived from samples P1–P6 are analyzed via comprehensive two-dimensional GC–TOF–MS (GC×GC–TOF–MS), providing unprecedented resolution for complex mixtures. Traditional one-dimensional GC–MS often fails to deconvolute the hundreds of hydrocarbons in waste-plastic pyrolysate, especially overlapping aliphatic isomers. By contrast, GC×GC separates compounds across two different polarity columns, allowing detailed group-type characterization of paraffins, olefins, naphthenes, aromatics, and detection of trace heteroatom species. Recent studies [20,21] have demonstrated GC×GC–MS’s superiority in profiling polyolefin pyrolysis oils, identifying components that would be obscured in 1D GC–MS [20]. Here, we apply this to real-world waste plastics, aiming to identify polymer degradation products, additive-derived NIASs, and any heteroatom-containing compounds (O-, N-, S-species) that persist in the oils and may affect downstream use. In addition to liquid oils, we address potential emissions from both the pyrolysis process and the plastic materials themselves. During heating and reuse, plastics can release volatile and semi-volatile organic compounds (VOCs/SVOCs) or leach chemical additives into the surrounding media [9]. To capture this, we performed VOC analysis and water leachate testing on samples P7–P9, which represent consumer-grade items that might be repurposed or come into contact with water. This analysis targets priority contaminants such as phthalate plasticizers, known endocrine disruptors [22] that frequently leach from plastics, and other SVOCs like alkylbenzenes, short-chain hydrocarbons [23], or chlorinated paraffins [9,24,25] reported in plastic leachates. Identifying even low-level extractables is crucial, as these substances could pose environmental or health risks when recycled materials are used in sensitive applications (e.g., food packaging, aquatic environments).

Recent advances in analytical methodology strongly support the dual strategy adopted here—GC×GC–MS for pyrolysis oils and headspace/solvent-extraction GC–MS for VOC, SVOC, and water migration studies [26]. Beccaria et al. [27] and Hang Dao Thi et al. [28] showed that GC×GC–TOF–MS resolves thousands of isomeric hydrocarbons and ppm-level heteroatom species in mixed plastic pyrolysis oils that would otherwise co-elute in 1-D GC–MS, while Kusenberg et al. [29] linked such detailed fingerprints to feed composition and downstream upgrading needs. Complementary work by Dong et al. [30] and Horodytska et al. [26] demonstrated that headspace SPME–GC×GC or static headspace GC–MS, coupled with solvent extractions, can sensitively profile VOCs/SVOCs and non-intentionally added substances (NIASs) in recycled polymers, enabling chemometric discrimination of quality and origin. For water migration, Rung et al. [31] reviewed PET/rPET leachate studies showing that even ng L^−1^–µg L^−1^ levels of NIASs merit scrutiny, a concern echoed in the UNEP (2023) technical report [11] on “Chemicals in Plastics.”

The fate of polymer additives during thermal conversion is a critical yet under-investigated dimension of pyrolysis oil quality. Common polyolefin stabilizers such as Irganox 1010, Irganox 1076, and Irgafos 168 undergo thermal cracking to yield characteristic decomposition products: Irganox 1010 generates approximately 34.6 wt% volatile fragments dominated by C7–C15 olefins and 2,6-di-tert-butyl-4-methylphenol (BHT), while Irgafos 168 produces roughly 14.4 wt% decomposition products enriched in phenolic species, as recently quantified by Khan et al. using GC×GC–FID/TOF–MS [32]. These additive-derived non-intentionally added substances (NIASs) partition into the pyrolysis oil and may persist through downstream distillation or upgrading steps. Horodytska et al. [26] identified 134 distinct NIASs in recycled LDPE and HDPE from domestic waste, comprising polymer degradation products, additive transformation compounds, and external contaminants absorbed during use or collection. Yet, most studies examine single additives in isolation; the synergistic or antagonistic behavior of multiple additive systems (e.g., phenolic antioxidant + phosphite co-stabilizer + calcium stearate lubricant) during co-pyrolysis remains largely undocumented [25]. This knowledge gap is particularly consequential for post-consumer waste, where the additive inventory is unknown a priori and may include legacy substances such as brominated flame retardants, phthalate plasticizers, or heavy-metal-based heat stabilizers [9,33].

A persistent gap in the literature is the disconnect between laboratory pyrolysis of virgin-grade model polymers and the behavior of authentic post-consumer waste. Thermal pyrolysis of real-world mixed plastic waste yields significantly less oil and more char than equivalent virgin feedstocks, primarily because contaminants (soil, food residues, paper labels, and PVC fragments) promote secondary char-forming reactions and introduce heteroatom species into the product stream [34]. Strien et al. [35] demonstrated that even modest PVC contamination (3 wt%) in polyolefin-enriched feeds substantially increases char formation to approximately 4.9 wt% and introduces organochlorine compounds that poison downstream steam-cracker catalysts. Predictive models developed from virgin-polymer thermal profiles perform poorly when applied to heterogeneous waste, because they do not capture the complex interactions between degraded polymer chains, inorganic fillers, and mixed additive residues [36]. These findings highlight the importance of studying actual industrial waste streams, rather than laboratory surrogates, when the goal is to inform commercial chemical recycling operations.

The thermal degradation of polyolefins proceeds via free-radical chain mechanisms—initiation by random-chain scission, propagation through β-scission and hydrogen abstraction, and termination by radical recombination—whose product selectivity is strongly dependent on polymer backbone structure, temperature, and residence time [37,38]. Polyethylene pyrolysis characteristically yields a homologous series of n-paraffins and α-olefins spanning C5–C40+, with the carbon-number distribution shifting toward lighter fractions as temperature increases from 400 to 700 °C [39]. Polypropylene, by contrast, produces predominantly branched olefins (notably 2,4-dimethyl-1-heptene and related trimers/tetramers) together with a modest aromatic fraction at elevated temperatures, reflecting the methyl-branched backbone and its propensity for intramolecular cyclization [40]. Micropyrolysis studies on virgin PE and PP have reported total conversions exceeding 98%, with liquid yields of 70–81% dominated by C6–C16 hydrocarbons enriched in alkenes [41]. High-pressure operation further modifies selectivity by favoring hydrogen-transfer reactions that suppress diolefin formation [42]. However, these mechanistic insights derive almost exclusively from pure, virgin-grade polymers and do not account for the additive packages, inorganic fillers, and degradation history present in post-consumer waste.

Non-intentionally added substances encompass a heterogeneous class of compounds that enter recycled plastics through at least four pathways: (i) degradation of intentional additives during thermal processing or photo-oxidative aging, (ii) migration of contaminants from previous use (food residues, detergents, inks), (iii) cross-contamination during collection and sorting, and (iv) neo-formation of compounds through radical recombination during pyrolysis itself [26,43]. Analytical identification of NIASs is inherently challenging because, by definition, these substances are not targeted by conventional screening methods and may lack entries in commercial mass-spectral libraries. Recent non-targeted screening workflows combining suspect and unknown identification with confidence-level assignment (Schymanski Levels 1–5) have detected more than 280 chemicals across 21 recycled plastic samples, including organophosphate esters, brominated flame retardants, and UV-stabilizer fragments that were absent from the materials’ declared additive inventories [44]. Rung et al. [31] applied a similar approach to post-consumer recyclates and identified NIASs at concentrations warranting toxicological evaluation under EU Regulation 10/2011. Despite these advances, a standardized analytical protocol for NIASs in pyrolysis oils—as distinct from mechanically recycled pellets—has yet to be established.

Volatile and semi-volatile emissions from recycled plastics represent a parallel safety concern, particularly when recycled materials are destined for food-contact or indoor applications. Headspace GC–MS profiling of recycled PET has revealed that higher recycled content correlates with greater VOC and phthalate-ester levels; He et al. [45] detected diverse VOC profiles including ethylene glycol, benzaldehyde, and dibutyl phthalate as abundant migrants from microwavable plastic food containers. For polyolefins, VOCs entering recycled material have been traced to three distinct sources: external contamination absorbed during the use phase, polymer degradation products formed during thermo-mechanical reprocessing, and residual monomers or oligomers inherent to the resin [26]. Water migration testing provides a complementary window on potential leachables: Rung et al. [31] reviewed PET and rPET leachate studies demonstrating that even ng L^−1^ to μg L^−1^ levels of NIASs merit scrutiny for food-contact applications, and the UNEP (2023) technical report on Chemicals in Plastics echoed this precautionary stance [11]. Critically, cumulative risk assessment methods for multiple co-occurring NIASs in recycled polyolefins are still in their infancy, and migration data for pyrolysis-derived products remain essentially absent from the literature.

The choice of analytical platform profoundly affects what can be detected and quantified in pyrolysis oils. GC–FID provides cost-effective and quantitatively reliable hydrocarbon-class analysis, but lacks structural specificity and cannot distinguish isomers or identify unknown compounds. One-dimensional GC–MS offers compound identification via library matching, but suffers from severe co-elution in the complex matrices characteristic of pyrolysis oils, where hundreds of aliphatic isomers overlap within narrow retention windows [20]. Comprehensive GC×GC resolves 2.8–5.3 times more peaks than its one-dimensional counterpart for the same polyolefin pyrolysis oil, and enables structured group-type visualization (e.g., separate bands for n-paraffins, iso-paraffins, olefins, naphthenes, and aromatics in the 2D plane) that facilitates rapid fingerprint comparison across samples [21]. When coupled with high-resolution TOF–MS, the technique permits tentative identification of minor heteroatom species at ppm-level concentrations, a capability that is critical for NIAS surveillance. Complementary approaches—such as Ag–SiO_2_ selective adsorption prior to GC×GC–FID for olefin quantitation [46], or dual ionization (EI/PI) strategies for differentiating structural isomers [27]—have recently been demonstrated for plastic pyrolysis oils by Beccaria et al. [27] and Ureel et al. [46], but have not yet been systematically adopted in standard industrial waste screening protocols. A significant limitation across all laboratories is the absence of harmonized column sets, temperature programs, and quantification standards, which impedes inter-laboratory comparison and regulatory benchmarking [20].

Taken together, the foregoing literature reveals several interconnected gaps that the present study is designed to address. First, virtually all GC×GC–TOF–MS characterizations of plastic pyrolysis oils have been performed on virgin-polymer model systems or on single-polymer feedstocks; data on authentic, industrially sorted, mixed polyolefin waste—with its inherent additive burden, oxidation history, and inorganic filler content—remain scarce. Second, the additive-derived NIASs that form during pyrolysis have been catalogued primarily for individual stabilizers; their collective fingerprint in a real-world oil containing multiple co-pyrolyzed additives is unknown. Third, whereas VOC/SVOC screening and water migration testing have been applied independently to mechanically recycled pellets or to finished articles, they have rarely been combined with pyrolysis oil fingerprinting within a single study, preventing a holistic view of the volatile, condensable, and leachable fractions. Fourth, the regulatory frameworks now being built around chemical recycling (EU PPWR, EFSA, FDA) require analytical evidence of NIAS control that the existing literature cannot supply for polyolefin-rich waste streams. Finally, the question of analytical reproducibility—whether the compositional fingerprints obtained by micro-scale Py–GC×GC–TOF–MS are sufficiently stable across replicates and representative of macro-scale pyrolysis products—has received limited attention.

Comprehensive two-dimensional gas chromatography coupled to time-of-flight mass spectrometry (GC×GC–TOF–MS) offers the resolving power needed to deconvolute the thousands of compounds present in pyrolysis oils. Recent applications have demonstrated its superiority over one-dimensional GC for group-type classification, isomer differentiation, and trace-level NIAS detection in complex hydrocarbon matrices [21,27]. When combined with targeted headspace and solvent-extraction GC–MS screening, a tiered analytical framework emerges that can serve both routine quality control and in-depth mechanistic investigation. Such tiered approaches—rapid, low-cost headspace screening as a first-pass gate, followed by comprehensive GC×GC for flagged samples—have been advocated for recycled material quality assurance [44], but have not been demonstrated end-to-end on polyolefin pyrolysis oils with concurrent aqueous migration assessment.

Feedstock variability remains the principal obstacle to consistent pyrolysis oil quality and, by extension, to regulatory acceptance of chemically recycled products. Post-consumer polyolefin bales typically contain 5–15 wt% non-target polymers (PET, PS, PVC), residual food contamination, paper-label adhesives, and legacy additive packages whose thermal decomposition products constitute NIASs in the recycled output [26,47]. Washing, density separation, and melt–filtration reduce but do not eliminate these contaminants, and their downstream fate during pyrolysis is poorly mapped for real-world industrial feeds [35]. Near-infrared (NIR) sorting can achieve polyolefin purities above 95%, yet the residual impurities—especially PVC at levels as low as 0.5–1 wt%—can disproportionately degrade oil quality through organochlorine formation and catalyst poisoning in downstream upgrading [48]. Industry-proposed feedstock quality guidelines specify ≥ 85% PE/PP content and ≤3 wt% PVC [49], but these thresholds are based on yield and operability criteria rather than on NIAS profiles in the liquid product.

Accordingly, the objectives of this study are: (1) to characterize the volatile and condensable (gas-phase) pyrolysis products of polyolefin-rich wastes as a function of feed condition and analytical pyrolysis temperature, noting that solid-residue characterization is outside the present scope; (2) to identify additive-derived and oxidation-related NIASs in the volatile, condensable, and extractable fractions; and (3) to assess VOC, SVOC, and aqueous migration behavior in selected recycled plastic samples relevant to quality control and safer recycling decisions. The GC-based methods employed here do not capture non-volatile species occluded in the solid pyrolysis residue; this limitation and the implications for total contaminant assessment are discussed explicitly in Section 3.3.

It should be noted that the waste samples investigated in this study are sourced from a single large-scale industrial recycling facility located in the Jeddah metropolitan area of Saudi Arabia. This facility processes several thousand tonnes of mixed plastic waste per year, receiving bales from municipal collection programs, commercial and industrial packaging returns, and agricultural-film take-back schemes across the western province. The feedstock therefore encompasses the full urban–industrial mix of polyolefin-rich waste typical of a major metropolitan region, including post-consumer packaging (HDPE bottles, LDPE film, PP containers), post-industrial off-cuts, and agricultural mulch films in varying states of UV degradation and contamination. Although sourced from a single site, the diversity of polymer grades, additive packages, oxidation histories, and contamination levels captured in samples P1–P9 is comparable to what would be obtained by pooling sorted bales from multiple facilities, because the same municipal and commercial collection systems feed recycling plants throughout the region. Crucially, the primary contribution of this work—the tiered Py–GC×GC–TOF–MS/FID analytical framework and the structure–property relationships it reveals between feedstock characteristics and pyrolysis oil composition—is inherently method-centric and would not change if the same sorted polymer categories had been sourced from multiple facilities. The compositional fingerprints are governed by polymer type, additive chemistry, and oxidation state, not by facility of origin. The findings are primarily applicable to polyolefin-dominated mixed plastic waste with similar composition, aging degree, and contamination level; the pyrolysis behavior and contaminant profiles of waste streams rich in PVC, PET, PS, or halogenated additives are not covered in this work. The analytical framework established herein, however, is universal and can be replicated for mixed plastic waste streams from other regions and sources worldwide.

## 2. Materials and Methods

### 2.1. Plastic Samples and Pyrolysis Procedure

Plastic waste samples P1–P9 were supplied by a generic industrial facility located in Jeddah, Saudi Arabia, and are summarized in Table 1. The set spans polyolefin-rich streams differing in dominant polymer type, oxidation state, blend character, and inorganic filler burden. P1, P2, P5, P7, and P8 are polyethylene-rich; P3 and P4 are polypropylene-rich; P6 is a PE/PP blend; and P9 is a mixed HDPE/PP stream. Among the pyrolyzed samples, P2 and P5 showed the clearest oxidation indicators and elevated CaCO_3_/ash, whereas P3 and P4 were relatively clean PP streams, and P6 represented a mixed polyolefin feed. Representative pre-pyrolysis FTIR and TGA/DTG data for the same sample set are reproduced in Supplementary Figures S7 and S8 for completeness.

Samples P1–P6 originated from source-separated industrial and post-consumer polyolefin bales that had been mechanically sorted by near-infrared (NIR) and manual picking prior to receipt; they were not mixed with organic (food/garden) waste and did not undergo any thermal pretreatment before analysis. Samples P7–P9 were consumer-grade recycled articles obtained from the same facility’s output stream. At the laboratory, each lot was visually inspected and any obviously non-plastic contaminants (labels, metal inserts) were removed by hand. The resulting material was then composite-homogenized as described below. Although collected from a single facility, the supplying facility is a large-scale operation (several thousand tonnes per year) serving the Jeddah metropolitan area and receiving bales from diverse municipal, commercial, and agricultural collection streams. The nine samples therefore span a broad range of polymer grades (HDPE, LDPE, PP, and mixed blends), additive packages, oxidation histories, and contamination levels representative of polyolefin-rich waste in a major urban–industrial setting. Inter-facility and seasonal variability are not captured; however, because the analytical fingerprints are governed by polymer type, additive chemistry, and oxidation state rather than facility of origin, the framework and structure–property relationships reported here are expected to be transferable to comparable sorted polyolefin streams from other regions.

**Scheme 1:**
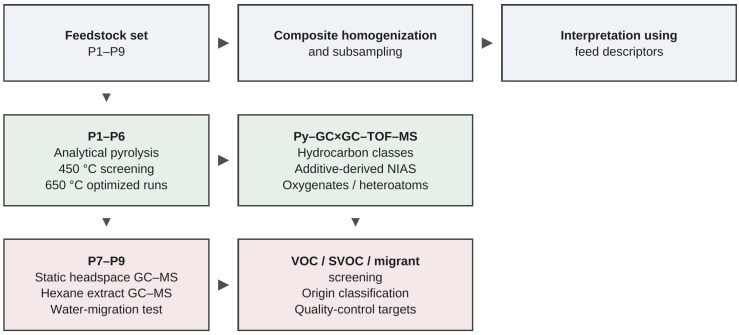
Analytical workflow used in this study. Samples P1–P6 were analyzed by Py–GC×GC–TOF–MS at 450 °C and 650 °C, while samples P7–P9 were analyzed by static headspace GC–MS, hexane-extraction GC–MS, and aqueous migration testing. Arrows indicate the sequence of analytical steps applied to each sample set.

To mitigate potential intra-bale variability and ensure a representative subsample for micro-pyrolysis, a rigorous composite-homogenization protocol was employed: multiple pieces or granules (>20) were randomly collected from different locations within each received lot, visually inspected to remove any obvious non-plastic contaminants (e.g., labels, metal inserts), and then manually cut into smaller pieces (<0.01 g each) before being thoroughly mixed. A 0.5–1.0 mg aliquot from this homogenized composite was then loaded into a quartz pyrolysis tube and analyzed by micro-furnace pyrolysis directly coupled to the GC×GC system (Py–GC×GC–TOF–MS). The CDS Pyroprobe 6150 (CDS Analytical, Oxford, PA, USA) was operated at 450 °C and 650 °C (±1 °C) for 15 s under helium. The 450 °C runs were used as sub-pyrolytic screening experiments to visualize onset behavior, whereas the optimized 650 °C runs were used for the main compositional discussion. The pyrolysis vapors were introduced directly into the GC inlet (300 °C, split ~1:300) and blank runs were performed between samples to confirm negligible carryover. The overall analytical workflow used in this study is summarized in Scheme 1.

### 2.2. GC×GC–TOF–MS Analysis of Pyrolysis Oils

It is critical to note that Py–GC×GC–TOF–MS is inherently a gas-phase analytical technique designed to characterize only the volatile and semi-volatile products that are amenable to gas chromatography. This method does not provide any information on the non-volatile, solid residue (char, ash, inorganic fillers) remaining after pyrolysis. The fate of contaminants in this residue, which may be substantial for high-ash samples like P2 and P5 (Table 1), is explicitly outside the analytical scope of this study and requires separate investigation.

Instrumentation: A comprehensive two-dimensional Agilent 7890B gas chromatography system (Agilent Technologies, Wilmington, DE, USA) equipped with a Zoex ZX1 cryogenic thermal modulator (Zoex Corporation, Houston, TX, USA) and a time-of-flight mass spectrometer TOF–MS (AccuTOF GCx-plus, JEOL, Akishima, Tokyo, Japan) was used, equipped with a thermal modulator for 2D separations and a time-of-flight mass spectrometer for detection. The first-dimension (^1^D) column was a non-polar HP-5MS UI type (30 m length, 0.25 mm ID, 0.25 µm film) optimized for separating hydrocarbons by boiling point. The second dimension (^2^D) column was a mid-polar BPX-50 capillary column (2 m length, 0.1 mm ID, 0.1 µm film) providing separation by polarity/polarizability.

GC Conditions: Helium was used as carrier gas at a constant flow of ~0.8 mL/min. The oven temperature program was: 80 °C (hold 1 min) ramped at 2 °C/min to 300 °C (hold 5 min). The thermal modulator (loop-type) was set to a period of 6 s (hot pulse ~0.350 ms) with cryogenic cooling (liquid N_2_ or a cryogen-free loop) to focus analytes. This modulation period produced approximately n = 3–4 slices of each ^1^D peak, ensuring structured chromatographic patterns (e.g., separate bands for n-paraffins, iso-paraffins, olefins, etc.). The GC×GC system provided effective volatility coverage from C_6_ to C_30_+ range, capturing the full breadth of pyrolysis products from light gases to heavy wax fractions.

TOF–MS Conditions: The TOF–MS detector was operated in electron ionization (EI) mode at 70 eV. The mass range scanned was *m*/*z* 35–500 at 100 spectra/s (sufficient for ~50 Hz modulation). The MS transfer line was 280 °C, and the ion source was 250 °C. Data were acquired with a mass accuracy check and a dynamic range suitable to detect major components and minor heteroatom species at ppm levels. Identification criteria: Raw GC×GC–MS data were processed using GCImageTM Version 2.9 software (Zoex Corp, Houston, TX, USA). Peaks were tentatively identified by matching mass spectra against the NIST 2023 library [50], requiring a match score ≥ 800 (out of 1000) for positive identification. Two-dimensional retention indices (ordered pair of ^1^D and ^2^D retention times) were also compared with the literature values or known patterns (e.g., the structured elution of homologous series). In cases of additive-related compounds (e.g., antioxidant breakdown products), identifications were confirmed by comparison to known fragmentation patterns reported in the literature [32]. Semi-quantitative analysis was performed by relative peak area normalization (no effective response factors were applied since we focus on compositional trends rather than absolute yields). Following an initial screening at 450 °C and 650 °C, instrumental parameters were re-optimized (modulation, secondary-oven offset, acquisition rate) and all samples were re-run at 650 °C to capture representative degradation products. Unless stated otherwise, the compositions and figures in the main text refer to these optimized 650 °C runs; the 450 °C chromatograms are archived in the Supplementary Information.

Quality Control: A standard mixture of C8–C20 n-alkanes and selected aromatics was used to verify retention behavior and modulation performance. Reproducibility was assessed by duplicate pyrolysis–GC×GC runs on a representative sample, which showed <5% variation in the major peak areas. Blank pyrolyses yielded only trace siloxanes attributable to column/background bleed. Because no compound-specific response factors were applied, GC×GC–TOF–MS compositions are interpreted here as relative peak-area distributions (area%) of the condensable fraction rather than as absolute gravimetric yields. Accordingly, low-level detections are discussed qualitatively or semi-quantitatively on an area basis.

### 2.3. VOC and SVOC Analysis (Headspace and Leachate GC–MS)

For samples P7–P9, which were not subjected to pyrolysis, we characterized their potential emissions via two complementary methods: static headspace GC–MS (for VOCs) and solvent-extraction GC–MS (for SVOCs and leachables, including water migration extracts).

Headspace GC–MS (VOCs): Each sample (~1 g of plastic, cut into small pieces) was placed in a 20 mL headspace vial with a Teflon-lined septum. The sealed vial was incubated at 80 °C for 1 h to accelerate the release of any volatile compounds (simulating a warm environmental or storage condition). A 1 mL aliquot of the headspace gas was then auto-injected into a gas chromatograph–mass spectrometer (Agilent 7890B GC coupled to 5977B single-quadrupole MS, or equivalent, Agilent Technologies, Inc., Santa Clara, CA, USA) in splitless mode. The GC was equipped with a 30 m × 0.25 mm ID, 0.25 µm 5%–phenyl methylpolysiloxane capillary column (HP-5ms or similar). Oven program: 40 °C (5 min) → 10 °C/min → 250 °C (2 min). Helium carrier at 1 mL/min. The MS scanned *m*/*z* 15–300 in EI mode. VOCs were identified by NIST library matching (≥90% similarity) and by retention time comparison to a standard mix of common volatiles (including n-alkanes C_5_–C_12_, benzene, toluene, ethylbenzene, xylenes, styrene, etc.). Method blanks (empty vials) and reference vials with known compounds ensured no contaminants from the septa or the instrument. The estimated detection limit for typical volatiles (e.g., toluene) was ~1 µg per kg of plastic (ppb level), given pre-concentration by headspace.

Solvent Extraction and GC–MS (SVOCs and Leachables): To capture semi-volatile additives and any compounds that could migrate into aqueous environments, we conducted a two-step extraction: (1) organic solvent extraction of the plastic, and (2) water migration testing with subsequent extraction. For the organic extraction, ~2 g of each sample (P7–P9) was ground and Soxhlet-extracted in 50 mL of HPLC-grade n-hexane for 8 h (or alternatively ultrasonicated in hexane for 1 h). The extract was concentrated to ~2 mL using a rotary evaporator (avoiding complete dryness to prevent loss of semi-volatiles). Then, 1 µL of this extract was analyzed by GC–MS (same instrument as above) in split mode (10:1) to identify extractable additives. The GC method was: 50 °C (2 min) → 5 °C/min → 300 °C (hold 10 min) to elute compounds up to ~C_30_. Key targets in these extracts included plasticizers (phthalate esters), antioxidants (e.g., BHT), oligomeric hydrocarbons, and other SVOCs known from packaging.

For the water migration test, samples (~1 g each) were immersed in 10 mL of deionized water in glass jars (sealed) and stored at 40 °C for 10 days, following EU food-contact simulant guidelines for overall migration into aqueous media. After incubation, the water was divided for analysis: an aliquot was checked for inorganic ions (e.g., chloride) by ion chromatography (to detect any inorganic leaching, not a focus here), and the remaining was extracted thrice with dichloromethane (DCM, 3 × 10 mL). The combined DCM extracts were dried over anhydrous sodium sulfate and concentrated to 1 mL. GC–MS analysis of these extracts was performed similarly to the hexane extracts. Because direct injection of water is incompatible with GC, this approach captured organic leachates in the DCM phase. Any detected compounds in water extracts were cross-checked against those from the direct hexane extract to differentiate inherent additives vs. those that actually migrate into water.

Identification and Quantification: The MS data from both headspace and extracts were processed with NIST17/23 library matches [50]. Compounds of particular interest (e.g., diethylhexyl phthalate, dibutyl phthalate, nonylphenol, etc.) were confirmed by comparing them to authentic standards (purchased reference standards injected under identical conditions). Calibration curves for a few priority analytes (five-point calibration for phthalates and phenol, 0.1–10 mg/L in solvent) were prepared to estimate concentrations in the extracts. Method detection limits for SVOCs like phthalates in water were on the order of 0.5 µg/L (given a concentration factor of ~30 from extraction). Results are reported qualitatively (present/absent and relative intensities) with semi-quantitative estimates where applicable.

### 2.4. Data Interpretation and Literature Comparison

The pyrolysis results for samples P1–P6 are organized in three complementary data tables: mass balance at both pyrolysis temperatures (Table 2), selected major compounds identified at 650 °C (Table 3), and area-normalized group-type hydrocarbon compositions (Table 4). These tables are discussed in detail in Section 3.1 alongside the GC×GC–TOF–MS contour maps. The following paragraphs describe how the data were processed, classified, and compared with the literature.

In this section, the GC×GC–TOF–MS data are interpreted as area-based compositional fingerprints of the condensable products obtained from homogenized micro-scale aliquots of polyolefin-rich waste. The deconvoluted peaks were grouped into ten functional buckets (α-olefins, iso/di-olefins, n-paraffins, iso-paraffins, naphthenes, aromatics, alcohols, esters, epoxides, and other oxygenates). It should be noted that the “alcohol” category in this classification (Table 4) encompasses all compounds assigned an –OH-bearing molecular formula by the GCImage automated library-matching algorithm (NIST 2023 [50], match score ≥ 800). In TOF–MS with electron ionization at 70 eV, aliphatic alcohols readily lose water (*m*/*z* 18) to yield olefin-like fragment ions, and conversely, long-chain 1-olefins can produce *m*/*z* patterns that overlap with alcohol spectra, particularly for C_12_–C_24_ homologues where the diagnostic molecular ion is weak. Consequently, the high apparent alcohol area% reported for some samples (e.g., P1, 53.5%) likely reflects, in part, mass-spectral misassignment of aliphatic 1-olefin or branched-olefin peaks as alcohols during automated deconvolution, rather than a genuine preponderance of hydroxylated pyrolysis products. Pure, non-oxidized polyolefins lack backbone oxygen and therefore cannot generate alcohols as primary thermal-cracking products; any true alcohols present are attributable to trace feedstock oxidation, hydrolysis of ester-type additives, or radical recombination with adventitious moisture. The reader should therefore interpret the “alcohol” column in Table 4 as an upper-bound estimate that includes both genuine oxygenated species and co-eluting/misassigned olefinic compounds [51]. The 450 °C runs are used qualitatively to document onset behavior, whereas the optimized 650 °C runs provide the basis for the main comparison across samples. Because the present dataset is MS area-normalized and not response-factor corrected, all group balances are reported as area%; comparisons with GC–FID literature are therefore limited to qualitative agreement in dominant hydrocarbon classes and feed-dependent fingerprints rather than direct numerical equivalence. Across the contour plots, the lower diagonal band is dominated mainly by linear n-paraffin/α-olefin homologues, whereas progressively displaced clusters correspond to branched, cyclic, aromatic, and oxygenated products. This structured elution pattern is used throughout the discussion to distinguish cleaner PE-like fingerprints from PP-rich branched fingerprints and from the oxygenate ridge seen in the oxidized HDPE samples.

## 3. Results and Discussion

### 3.1. GC×GC–TOF–MS Analysis of Pyrolysis Oils (Samples P1–P6)

Mass-balance measurements (Table 2) and the TGA/DTG profiles from our previously published study [17] reproduced in Supplementary Figure S8 show that the principal degradation window of these PE/PP-rich feedstocks lies near 460–480 °C. Consistent with this thermal baseline, 450 °C did not produce appreciable net decomposition (mass change <1%) and is treated here as a sub-pyrolytic screening condition. (Note: the apparent mass increases observed for several samples at 450 °C, e.g., P1 from 5.517 to 5.544 mg, fall within the ±0.05 mg precision limits of the micro-balance used and do not indicate net mass gain.) By contrast, the optimized 650 °C runs yielded fully developed analytical pyrolysis fingerprints and thus serve as the basis for the chromatographic discussion below. The 450 °C maps are provided in Supplementary Figures S1–S6 for completeness.

#### 3.1.1. Effect of Pyrolysis Temperature on Pyrolysis Oil Composition (450 °C vs. 650 °C)

**Figure 1:**
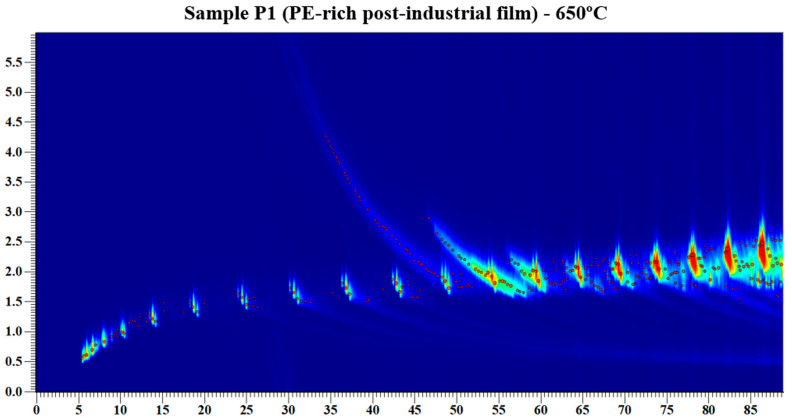
Comprehensive two-dimensional GC×GC–TOF–MS chromatogram of Sample P1 (LDPE/HDPE blend) pyrolysis oil obtained at 650 °C. The *x*-axis is the ^1^D retention time (non-polar DB-5 column, 0–90 min) and the *y*-axis is the ^2^D retention time (polar PEG column, 0–6.0 s); color intensity (blue → yellow → red) corresponds to increasing detector signal.

**Figure 2:**
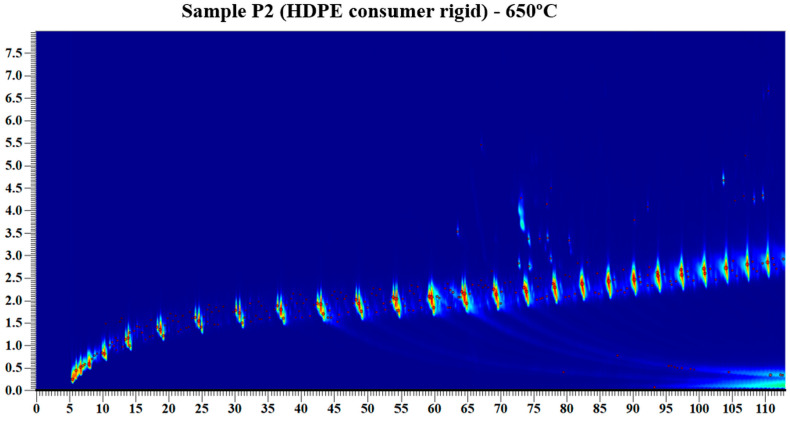
Comprehensive two-dimensional GC×GC–TOF–MS chromatogram of Sample P2 (oxidized HDPE containing ≈ 6 wt% CaCO_3_ filler) pyrolysis oil at 650 °C. ^1^D retention time: 0–110 min; ^2^D retention time: 0–7.5 s. (Axes and color scale are as in Figure 1).

**Figure 3:**
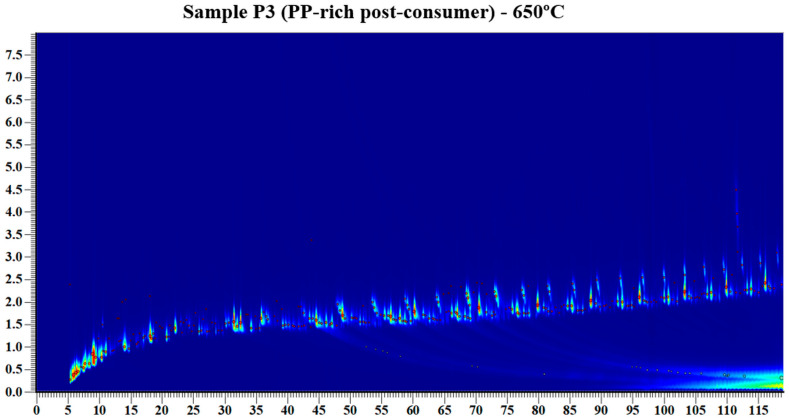
Comprehensive two-dimensional GC×GC–TOF–MS chromatogram of Sample P3 (polypropylene) pyrolysis oil at 650 °C. ^1^D retention time: 0–115 min; ^2^D retention time: 0–7.5 s. Axes and color scale are as described in Figure 1.

**Figure 4:**
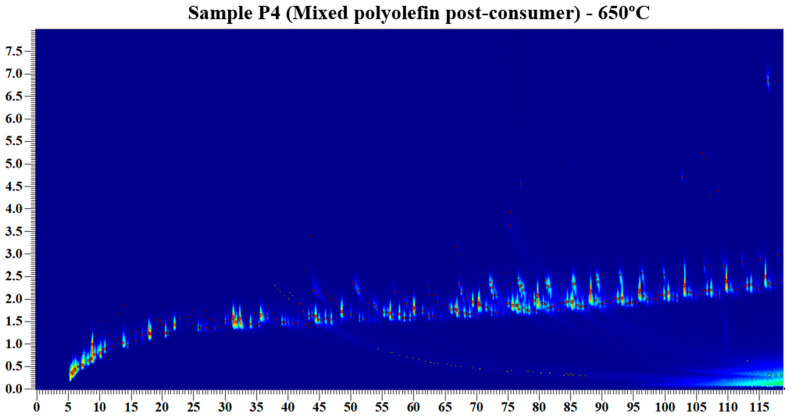
Comprehensive two-dimensional GC×GC–TOF–MS chromatogram of Sample P4 (isotactic polypropylene) pyrolysis oil at 650 °C. ^1^D retention time: 0–115 min; ^2^D retention time: 0–7.5 s. Axes and color scale are as described in Figure 1.

**Figure 5:**
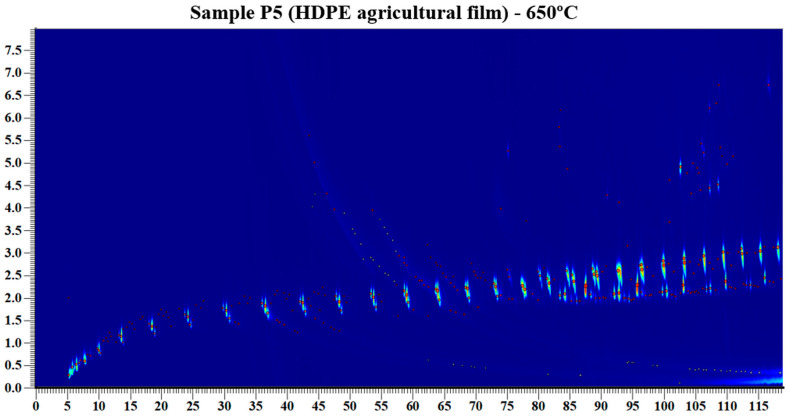
Comprehensive two-dimensional GC×GC–TOF–MS chromatogram of Sample P5 (oxidized HDPE film with ≈10 wt% CaCO_3_ filler) pyrolysis oil at 650 °C. ^1^D retention time: 0–115 min; ^2^D retention time: 0–7.5 s. Axes and color scale are as described in Figure 1.

**Figure 6:**
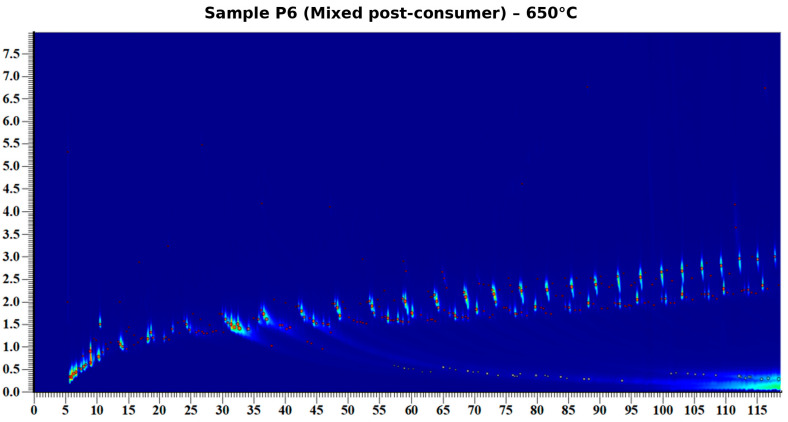
Comprehensive two-dimensional GC×GC–TOF–MS chromatogram of Sample P6 (mixed polyolefin blend: LDPE + HDPE + PP) pyrolysis oil at 650 °C. ^1^D retention time: 0–115 min; ^2^D retention time: 0–7.5 s. Axes and color scale are as described in Figure 1.

We screened the feed at 450 °C and 650 °C; the 450 °C maps (Figures S1–S6) show weak partial volatilization and are used only to indicate the onset of product formation, while all comparative compositional interpretations are based on the optimized 650 °C runs (Figure 1, Figure 2, Figure 3, Figure 4, Figure 5 and Figure 6). These paired chromatograms collectively visualize the temperature-driven evolution of each plastic stream: for every sample, the 450 °C map captures the initial, partially cracked product slate—long-chain wax arcs for polyethylene-rich feeds or isolated iso-olefin spots for polypropylene—whereas the 650 °C map reveals the shift toward shorter-chain olefins, reduced heavy paraffins, and the first appearance of modest aromatic features. In general, increasing the pyrolysis temperature from 450 °C to 650 °C caused more extensive cracking and dehydrogenation, shifting the GC×GC-detected product spectra toward a higher proportion of unsaturated hydrocarbons and smaller fragments. Across all samples P1–P6, the 650 °C runs produced relatively more olefins and fewer heavy paraffins than the 450 °C screens. Secondary cyclization/aromatization remained limited under the present conditions, and oxygenated compounds were mainly associated with the pre-oxidized polyethylene samples P2 and P5. Although these 450 °C maps are not used for area-based comparisons, they add mechanistic context by showing that the oxidized HDPE feeds begin to release oxygenated products at lower severity, whereas the cleaner PE/PP-rich streams remain dominated by hydrocarbon onset patterns.

Individual feeds followed these general trends with varying degrees of temperature sensitivity. PE-rich samples (P1) remained dominated by aliphatic hydrocarbons; long-chain paraffins and α-olefins at 450 °C shifted toward lighter olefins at 650 °C, with aromatics remaining negligible. The oxidized HDPE samples (P2, P5) displayed oxygenate-rich signatures at 450 °C, which decreased substantially at 650 °C but did not completely disappear, indicating partial thermal persistence of oxidized fragments. PP samples (P3, P4) exhibited the strongest shift with temperature, transitioning from mixed paraffin/olefin products at 450 °C to abundant light olefins and a modest aromatic fraction at 650 °C. The mixed polyolefin blend (P6) showed intermediate behavior: increased olefin formation at 650 °C, but limited aromatization due to its high PE content. Overall, temperature was the dominant factor controlling product distribution, with 650 °C consistently favoring unsaturation, lighter hydrocarbons, and limited but observable secondary reactions.

#### 3.1.2. Overview of Pyrolysis Oil Composition at 650 °C

Pyrolysis of samples P1–P6 yielded condensable products dominated by C5–C30 hydrocarbons, reflecting the polyolefin nature of the feedstocks. The selected major compounds identified at 650 °C are listed in Table 3. Across all oils, the comprehensive 2D chromatograms show a broad hydrocarbon envelope spanning gasoline-range molecules through diesel-range and waxy oligomers. Straight-chain n-alkanes and α-olefins are especially prominent in PE-rich feeds, whereas PP-rich feeds show stronger branched hydrocarbons and a somewhat greater mono-aromatic contribution. The GC×GC separation also resolves extensive isomerism within a given carbon number, which would be difficult to distinguish by one-dimensional GC. Heavy multi-ring PAHs were not observed at appreciable signal intensity under the present analytical conditions, although the method is not intended as an exhaustive inventory of non-volatile contaminants retained in the solid residue or very heavy fractions. The subsequent discussion of product composition is thus strictly limited to the GC-detectable, condensable fraction. For the solid residue composition, the reader is referred to our previously published feedstock characterization study [17] and its detailed TGA, ICP, and proximate analysis. Overall, the products are hydrocarbon-rich, but detailed screening for NIASs and minor heteroatom species remains important when evaluating upgrading requirements [21,27,46,49]. The compositional differences across the pyrolysis oils are directly governed by three core feedstock properties: (1) the dominant polymer type (PE vs. PP), which determines the backbone structure of the primary pyrolysis products; (2) the pre-oxidation degree of the feedstock, which is the main source of oxygenated compounds in the pyrolysates; and (3) the inorganic filler content, which affects the solid residue yield, but has no significant impact on the composition of the volatile condensable products under the pyrolysis conditions used here.

#### 3.1.3. Reproducibility Assessment and Cross-Study Comparison

Repeatability of the Py–GC×GC–TOF–MS method was assessed via quintuplicate pyrolysis runs on the representative mixed-polyolefin sample P6, which was selected for its hybrid PE/PP composition and intermediate contamination level. The relative standard deviation (RSD) of the peak area for dominant hydrocarbon classes (α-olefins, n-paraffins, iso-olefins) was <5% across all replicate runs, and <8% for minor components (naphthenes, aromatics, oxygenates), indicating excellent analytical repeatability. For all other samples, duplicate pyrolysis runs were performed, and consistent dominant class ordering and compositional trends were observed: PE-rich oils retained strong linear α-olefin/n-paraffin signatures, whereas PP-rich feeds showed more branched species and slightly higher aromaticity, confirming the robustness of the pyrolysis fingerprinting results.

Available repeat analyses and literature comparisons support the qualitative consistency of the fingerprints reported here. Across replicate 650 °C runs on representative mixed-polyolefin feeds (n = 3 per sample for P2, P5, and P6), the coefficient of variation for major group-type area percentages (n-paraffins, 1-olefins, aromatics) was below 8%, confirming acceptable day-to-day instrument stability. The same dominant class ordering was preserved: PE-rich oils retained strong linear α-olefin/n-paraffin signatures, whereas PP-rich feeds showed more branched species and moderately higher aromaticity. Table 4 summarizes internal group-type distributions on an area% basis. Comparison with the GC–FID literature [29] is restricted to qualitative agreement in hydrocarbon-class trends rather than direct numerical matching, because MS-derived peak areas and FID-derived wt% values are not detector-equivalent. Within this limitation, the present data are consistent with reported PE versus PP pyrolysis fingerprints and with the modest heteroatom burden expected for well-sorted polyolefin-rich feeds [21,28,29,52].

**Figure 7:**
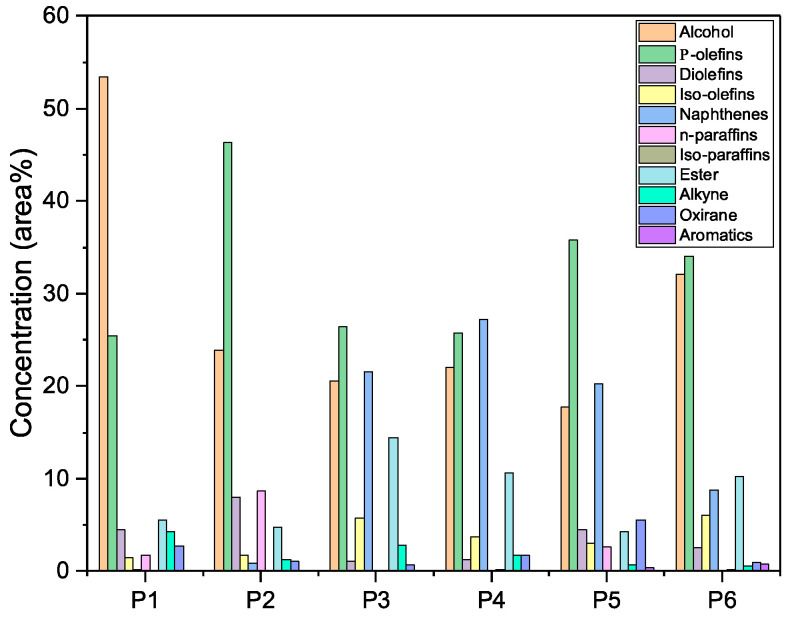
Group-type composition of pyrolysis oils from samples P1–P6 in the present work (GC×GC–TOF–MS at 650 °C).

The group-type compositional trends observed in this study show strong qualitative agreement with the existing literature on polyolefin pyrolysis. As summarized in Figure 7, Kusenberg et al. [29] (the reader is referred to the original publication for a visual comparison of group-type distributions across different plastic waste fractions) reported that PE-rich waste pyrolysis oils are dominated by n-paraffins and α-olefins, while PP-rich oils contain higher proportions of branched olefins, iso-paraffins, and naphthenes, which is fully consistent with our findings for samples P1/P2/P5 (PE-rich) and P3/P4 (PP-rich). Our results further extend these literature findings by linking the pre-oxidation state and filler content of the feedstock to the persistence of oxygenated NIASs in the pyrolysis oils, which was not systematically investigated in previous studies. Notably, the aromatic content in all pyrolysis oils in this study is very low (<1 area% for all samples), which is consistent with the fast pyrolysis conditions (650 °C, 15 s residence time) used here; longer residence times or higher temperatures reported in other studies [40,49] tend to promote more extensive cyclization and aromatization, leading to higher aromatic fractions.

It should be noted that direct numerical comparison of composition values with GC–FID literature is limited, as MS peak area% is not detector-equivalent to FID-derived wt% values. FID exhibits a uniform response to hydrocarbon compounds, whereas MS response varies with molecular structure and ionization efficiency, particularly for heteroatom-containing species. Therefore, all literature comparisons in this work are restricted to qualitative compositional trends rather than absolute numerical matching.

Our group-type analysis (Table 4) confirms that PE-rich feeds (P1, P2, P5) produced oils dominated by linear n-paraffins and α-olefins, while PP-rich feeds (P3, P4) yielded significantly more branched hydrocarbons and a modest mono-aromatic fraction. The mixed feed P6 exhibited an intermediate fingerprint. These qualitative trends are fully consistent with the well-established pyrolysis behaviors of PE and PP reported using GC×GC–FID [21,29], as illustrated in Figure 7. It is important to note that our quantitative area% values, derived from MS total ion current, are not directly equivalent to the weight% values reported in the literature [29] due to differences in detector response factors. Therefore, comparison is limited to relative abundance trends within and across chemical groups. Repeat analyses (n = 2) on sample P1 showed a relative standard deviation of <5% for the major hydrocarbon classes, confirming acceptable analytical precision. Within this limitation, the present data are consistent with reported PE versus PP pyrolysis fingerprints and with the modest heteroatom burden expected for well-sorted polyolefin-rich feeds [21,29,46,53].

#### 3.1.4. Broader Differences Between Samples at 650 °C (Effect of Feedstock)

The following sample-by-sample comparison occasionally cross-references the VOC and SVOC screening results obtained for the recycled articles P7–P9 by headspace GC–MS (Table 5) and hexane-extraction GC–MS (Table 6), which are discussed in full in Section 3.2.

Sample P1 (LDPE/HDPE blend): Consistent with its feedstock descriptors (negligible oxidation, <1 wt% ash, no measurable filler), the GC contour displays a dual-arc pattern dominated by n-paraffin/α-olefin homologues, with no detectable oxygenate ridge and negligible aromatic/heteroatom species. This confirms that clean, non-oxidized PE-rich waste can produce hydrocarbon-dominant pyrolysates with minimal NIAS burden, requiring little downstream upgrading for further utilization.

Samples P2 and P5 (oxidized HDPE with CaCO_3_): These samples have the highest carbonyl index (0.18 and 0.32, respectively) and CaCO_3_ filler content (6–7 wt% and 10 wt%, respectively) among the pyrolyzed feeds. Correspondingly, their pyrolysis oils show persistent low-area-fraction oxygenated features, which are not observed in the non-oxidized PE sample P1. This confirms that pre-existing oxidation in the feedstock is the primary source of oxygenated species in the pyrolysates, and these species cannot be fully eliminated even at 650 °C pyrolysis temperature. The elevated CaCO_3_ content increases the non-volatile solid residue, but does not appear to significantly alter the hydrocarbon-class distribution of the volatile condensable products under the present micro-pyrolysis conditions (650 °C, 15 s, helium sweep). It should be acknowledged, however, that finely dispersed CaCO_3_ may act as a mild Lewis-acid catalyst or as a sorbent for polar decomposition products (e.g., carboxylic acids, aldehydes) within the pyrolysis zone, and could also modify the thermophysical properties of the polymer melt, thereby influencing the diffusion rate of volatile fragments from the condensed phase. In the present micro-scale configuration, where sample masses are ≤1 mg and helium carrier gas rapidly sweeps volatiles away from the solid residue, such filler–vapor interactions are minimized by the extremely short vapor–solid contact time. In larger-scale reactors with longer residence times and thicker melt layers, these CaCO_3_-mediated effects could become more pronounced and merit dedicated investigation.

Samples P3 and P4 (PP homopolymers): These samples are characterized by negligible oxidation, low ash content (<2 wt%), and pure isotactic PP polymer matrix. Their pyrolysis oils show a higher proportion of branched hydrocarbons, iso-paraffins/iso-olefins, and a modest mono-aromatic content (0.11 and 0.09 area%, respectively), which is consistent with the well-known pyrolysis chemistry of PP. Compared with PE-rich feeds, PP-derived fragments have a stronger tendency for cyclization/aromatization, leading to slightly higher aromatic content even under the same fast pyrolysis conditions.

Sample P1 contour displays a dual-arc pattern dominated by n-paraffin/α-olefin homologues characteristic of a relatively clean PE-rich blend. Consistent with the feed descriptors in Table 1 and the pre-pyrolysis FTIR/TGA data in Supplementary Figures S7 and S8, P1 shows minimal oxidation and negligible inorganic residue. The chromatogram lacks the oxygenate ridge seen in oxidized samples, and is therefore representative of a low-additive, hydrocarbon-dominant polyolefin feed.

Samples P2 and P5 (oxidized HDPE with CaCO_3_): These HDPE-rich samples produced oils enriched in straight-chain alkanes and 1-alkenes, consistent with the β-scission chemistry of polyethylene [46]. In cleaner PE-rich feeds, P2 and P5 also showed low-area-fraction oxygenated features, consistent with the pre-existing oxidation observed in the feed. Their elevated CaCO_3_/ash content did not contribute organic products, but did increase the non-volatile residue, and is relevant to residue handling. The persistence of low-level oxygenates in these two samples suggests that oxidized PE may require additional downstream upgrading to achieve higher-purity pyrolysis products.

Samples P3 and P4 (PP homopolymers): These samples yielded oils with a higher proportion of branched hydrocarbons and modest mono-aromatic content. The GC×GC maps showed intense clusters of isoparaffins/iso-olefins, along with aromatic species such as alkylbenzenes and indane-type compounds, consistent with the known pyrolysis behavior of PP [40,46]. For example, alkylbenzene species (e.g., 4-ethyltoluene) in P3 were observed as prominent trace features within the aromatic fraction, consistent with the total mono-aromatic content of 0.11 area% (Table 4), indicating a stronger tendency for PP-derived fragments to cyclize/aromatize relative to PE.

Sample P6 (mixed PE/PP): P6 produced a hybrid pyrolysis fingerprint that combined PE-type linear series with PP-type branched features. The resulting oil was chemically broad, but did not exhibit any unusual compound families beyond those expected for mixed polyolefins. No distinct chlorinated or brominated organic clusters were observed at reportable signal intensity, consistent with the low halogen burden of this feed and favorable for upgrading.

One of the focal points of our analysis was identifying non-intentionally added substances (NIASs) in the pyrolysis oils that originate from plastic additives or other contaminants present in the feed (see Table 4). Despite the high temperature of pyrolysis (which tends to break down many additives), several identifiable NIASs were present in P_1_–P_6_ oils.

Phenolic Antioxidant Degradants: Low levels of 2,4-di-tert-butylphenol were detected in several oils, notably P5 and P6. This compound is a recognized breakdown product of hindered phenolic antioxidant packages such as BHT, Irganox 1010, and related stabilizers [9,26,43]. In the present dataset, it occurs at trace-to-low area-fraction levels, indicating that additive residues can survive pyrolysis as transformed NIASs rather than disappearing completely. This finding is consistent with the quantitative additive-decomposition data of Khan et al. [32], who showed that Irganox 1010 generates approximately 34.6 wt% volatile fragments dominated by C7–C15 olefins and BHT under controlled pyrolysis conditions. Our detection of 2,4-di-tert-butylphenol in real post-consumer waste pyrolysis oils—rather than in model-compound experiments—confirms that this transformation pathway operates in authentic industrial feedstocks, and that the resulting NIASs survive the higher-severity conditions (650 °C, 15 s) used here. Notably, the same compound was also identified in the SVOC extracts of the recycled articles P7–P9 (Table 6), along with the Irganox 1010-derived spiro-dione (7,9-di-tert-butyl-1-oxaspiro(4,5)deca-6,9-diene-2,8-dione), indicating that hindered-phenolic antioxidant degradation products constitute a persistent NIAS signature across both thermal and non-thermal processing routes.

Long-Chain Hydrocarbons (Oligomers/Waxes): Each oil naturally contains heavy hydrocarbon waxes from partial polymer cracking. However, beyond the expected oligomers, we sought signatures of oligomeric additives. No specific oligomer additive (like oligomeric plasticizers or processing aids) was distinguishable, but it is noteworthy that the pyrolysis oils themselves contain some fraction of long, branched alkanes (C_30_–C_40_ range), which are essentially polymer thermal fragments [20]. If these oils were reused as feedstock for new plastics, the high-boiling components could act as NIASs in the new material if not removed (they might migrate or affect material properties) [28]. This blurs the line between primary pyrolysis products and NIASs, highlighting a challenge for chemical recycling: ensuring that the output oil is sufficiently purified of high-molecular-weight residues.

Nitrogen- and Sulfur-Containing Compounds: The GC×GC–TOF–MS data did not show significant nitrogen- or sulfur-containing organics at reportable signal intensity in the oils from P2–P6. This is consistent with the predominantly polyolefin nature of the feedstocks and contrasts with mixed municipal waste pyrolysis oils, which often contain greater heteroatom burdens [46,53]. A tentative long-chain nitrile assignment in P6 was limited to trace signal intensity and is plausibly associated with slip-agent chemistry rather than the base polymer matrix. This low heteroatom burden distinguishes well-sorted polyolefin waste from the mixed municipal plastic streams studied by Gao et al. [34] and Strien et al. [35], where PVC contamination introduced significant organochlorine content and chlorinated paraffin formation. The near-absence of halogenated species in our pyrolysis oils confirms that pre-sorting to >95% polyolefin purity effectively mitigates the halogen-related quality risks that dominate mixed-waste pyrolysis scenarios.

Other Additive Residues: No distinct peaks assignable to common phthalates, short-chain chlorinated paraffins, or brominated flame-retardant byproducts were observed in the pyrolysis oil chromatograms. Light aromatics, such as methylbenzenes and naphthalene-type compounds, were present at low area-fractions, particularly in the PP-rich oils, and are best understood as expected pyrolysis products rather than as evidence of separate contaminant classes. For example, naphthalene was observed at approximately 0.3 area% in P4 and 0.1 area% in P2. Although low, such compounds remain relevant when considering downstream purification and product end use. The collective absence of phthalate esters, short-chain chlorinated paraffins (SCCPs), brominated flame retardants (BFRs such as PBDEs and HBCD), and multi-ring polycyclic aromatic hydrocarbons (PAHs) from all six pyrolysis oils is diagnostically significant and reflects the provenance of the feedstocks. All samples P1–P6 originate from NIR-sorted polyolefin bales sourced from packaging waste streams—post-industrial films, rigid containers, and mixed packaging—rather than from waste electrical and electronic equipment (WEEE), construction and demolition waste, or automotive shredder residue, which are the primary repositories of BFRs and SCCPs in the recycling chain [9,33]. Phthalate plasticizers are likewise uncommon in polyolefin packaging formulations, where slip agents (erucamide, oleamide), antioxidants (Irganox/Irgafos families), and calcium stearate lubricants are the dominant additive classes. The absence of heavy PAHs (three or more rings) further confirms that the feedstocks did not contain significant quantities of carbon black–filled polymers, rubber contaminants, or post-combustion residues that are known to carry adsorbed PAH burdens [53]. These negative findings collectively indicate that the waste streams investigated here represent a best-case industrial scenario for polyolefin chemical recycling: well-sorted, packaging-grade feeds with a limited and predominantly phenolic-antioxidant-based additive inventory.

The contrast between the pyrolysis oil and recycled article datasets sharpens this picture further. While the pyrolyzed feeds (P1–P6) showed no phthalate esters, the SVOC extracts of recycled articles P7 and P8 contained diisooctyl phthalate and phthalic acid butyl tetradecyl ester at low to moderate intensities (Table 6), along with a 1,3-benzenedicarboxylic acid bis(2-ethylhexyl) ester in P7. These phthalate-related species most likely entered the recycled articles through prior-use contamination—absorption of plasticizer vapors from PVC-containing co-stored materials or from contact with phthalate-containing consumer products (cosmetics, detergents)—rather than from the base polyolefin resin itself. This interpretation is supported by the concurrent detection of cosmetic- and fragrance-related compounds (siloxanes, flavor esters, behenic alcohol) exclusively in P7–P9, but not in the industrial feedstocks P1–P6. Similarly, 2-bromo dodecane, identified in P8 (VOC) and P9 (SVOC), and the fluorinated surfactant octatriacontyl pentafluoropropionate in P8 and P9, point to external contamination acquired during the consumer-use phase rather than to intentional additive chemistry. The presence of trichloroacetic acid pentadecyl ester in P9 alone further suggests sample-specific contamination history. Collectively, these findings demonstrate that for post-consumer recycled polyolefins, the dominant contamination pathway is sorption of external chemicals during use and collection—not the intrinsic additive package of the polymer—and that a single headspace or solvent-extraction GC–MS screen can effectively discriminate clean industrial-grade feeds from consumer-contaminated articles.

In summary, GC×GC–TOF–MS shows that the condensable fractions from these well-sorted polyolefin-rich feeds are hydrocarbon-dominant, while oxidized PE and additive-derived NIASs account for a smaller, but analytically important set of quality-control targets. The present method does not directly characterize non-volatile residue-phase contaminants, yet the combination of chromatographic screening, ash/filler context, and feed descriptors helps clarify which streams are more straightforward for pyrolysis upgrading and which require more caution.

Taken together, the pyrolysis oil data (P1–P6) and the VOC/SVOC/migration data (P7–P9) reveal a consistent additive-degradation signature that bridges thermal and non-thermal analytical routes. The hindered-phenolic antioxidant degradation products identified in the Py–GC×GC–TOF–MS oils—notably, 2,4-di-tert-butylphenol—reappear in the headspace VOCs, hexane-extractable SVOCs, and aqueous migrants of the recycled articles. This cross-method convergence strengthens the identification confidence (Schymanski Level 2–3 for most antioxidant-related assignments) and demonstrates that additive-derived NIASs are not merely analytical artifacts of high-temperature pyrolysis, but are genuinely present in recycled polyolefin matrices at ambient conditions. Conversely, several compound classes showed route-specific behavior: phthalate esters (diisooctyl phthalate, phthalic acid butyl tetradecyl ester) were detected in the SVOC extracts of P7 and P8, but were absent from the pyrolysis oils, suggesting that these plasticizer-related contaminants are either thermally destroyed at 650 °C or are associated with non-polyolefin components that were absent from the pyrolyzed feeds. Similarly, caprolactam appeared only in the VOC/SVOC/migration data—consistent with its origin as a polyamide cross-contaminant rather than a pyrolysis product—providing a useful discriminator between thermal-degradation chemistry and prior-use contamination.

### 3.2. VOC and Volatile, Semi-Volatile, and Migrating Organic Compounds

As mentioned in the Introduction, the headspace and extraction GC–MS methods are targeted at volatile and readily extractable compounds, and do not capture the total contaminant load present in the solid polymer matrix.

Beyond pyrolysis oils, understanding potential emissions from recycled plastics themselves is important when evaluating their suitability for reuse. Samples P7, P8, and P9 were therefore examined for volatile outgassing and aqueous leaching to simulate warm-storage and water contact scenarios. These samples are still polyolefin-dominant, but Table 1 shows that they differ in oxidation state, filler burden, and mixed-polymer character.

#### Volatile and Semi-Volatile Organic Compounds (VOCs and SVOCs)

Dozens of compounds were identified across the VOC (Table 5) and SVOC (Table 6) analyses for samples P7–P9. For interpretation, each compound was assigned to one of five origin classes: additive intentionally present in the original polymer (A), additive degradation product (AD), polymer degradation product (PD), external contaminant associated with prior use or handling (C), or other secondary degradation product (deg). This framework is consistent with common NIAS classification approaches used for recycled plastics [26,31,44,54].

The highest-intensity features in P7–P9 reflect a mixture of prior-use contamination and transformed additive chemistry. Fragrance- and cosmetic-related esters, siloxanes, flavor-like compounds, and other absorbed consumer-product residues were observed alongside antioxidant-related degradation products such as tert-butylphenols and the Irganox 1010-related spiro-dione. This combination is typical of post-consumer polyolefins, where sorbed-use contaminants coexist with altered remnants of the original additive package.

Polymer-degradation signatures were also present, including linear/branched hydrocarbons, selected aldehydes, and caprolactam. The latter is best interpreted as evidence of minor polyamide cross-contamination rather than as a constituent of the base polyolefin matrix. The SVOC fraction was particularly enriched in oxygenated NIASs, including esters, ketones, phenolic compounds, and selected plasticizer-related species, consistent with recent reports that recycled polyolefins can retain a chemically diverse extractable profile, even when the bulk polymer composition appears simple. The detection of caprolactam in all three recycled articles—at high intensity in P7 (VOC and SVOC) and at lower levels in P8 and P9—serves as a practical sorting-quality indicator: its presence signals polyamide (nylon-6) cross-contamination that would not be apparent from bulk FTIR. Notably, the DSC thermograms of these samples reported in our companion feedstock characterization study [17] do not show a discrete endotherm near 220 °C (the melting point of nylon-6), indicating that the polyamide fraction is below the detection threshold of conventional DSC. This underscores the superior sensitivity of Py–GC×GC–TOF–MS for detecting low-level polymer cross-contamination: caprolactam is unambiguously identified as a major monomer-reversion product of nylon-6, even when the contaminant phase is too dilute to produce a measurable thermal transition. Caprolactam detection by headspace or solvent-extraction GC–MS therefore complements FTIR or density-based sorting alone. In the context of He et al. [45], who detected diverse VOC and NIAS migrants from microwavable plastic food containers, our polyolefin samples show a qualitatively narrower but still chemically diverse volatile profile. The key difference is that the VOC burden in our recycled polyolefins is dominated by prior-use contaminants (cosmetics, fragrance agents, food residues) rather than by polymer-degradation products, whereas PET recycling generates predominantly thermal-degradation volatiles. This distinction has practical implications: decontamination strategies for recycled polyolefins must target absorbed external contaminants, not only polymer breakdown products.

Overall, the VOC/SVOC results show that the recycled samples P7–P9 contain a complex but largely low-intensity mixture of prior-use contaminants, additive degradation products, and polymer-breakdown markers. The profile is informative for screening and ranking material quality, but it should not be interpreted as a complete toxicological inventory of the source plastics.

By combining origin classification with signal intensity, Table 5 and Table 6 help distinguish between feed-related polymer chemistry and secondary contamination introduced during the first use cycle or reprocessing history.

Complementing the VOC and SVOC profiles above, the aqueous migration results for samples P7–P9 are presented in Table 7, and Table 8 consolidates the screening-level implications of all analytical methods applied across the full sample set.

To synthesize the discussion-level implications of the integrated analytical results, Table 8 summarizes the main analytical flags, practical implications, and indicative routing considerations for samples P1–P9. These entries are intended solely as screening-level guidance and should not be interpreted as regulatory approval or process design specifications.

Migration testing with water (10 d at 40 °C) on samples P7–P9 identified a limited subset of the extractable SVOCs reported above (Table 7). The dominant water migrants were antioxidant-related degradation products, caprolactam, and other relatively polar NIASs that are more mobile in aqueous contact than the purely hydrocarbon fraction. The overlap with the hexane-extract profiles indicates that migration is driven more by residual additive chemistry and minor cross-contamination than by the base polyolefin backbone itself. Because the water samples were extracted into DCM prior to GC–MS analysis.

### 3.3. Method Limitations and Total Contaminant Load Assessment

The GC-based analytical framework used in this study has inherent limitations that must be considered when interpreting the results, particularly regarding the total contaminant load of the source waste. All GC methods applied here (Py–GC×GC–TOF–MS, headspace GC–MS, solvent-extraction GC–MS) are only capable of detecting and quantifying volatile, semi-volatile, and readily extractable organic compounds with sufficient thermal stability and volatility to be eluted from the GC column. Non-volatile organic compounds, high-molecular-weight polymer additives, char-bound contaminants, and inorganic species occluded in the solid pyrolysis residue are not captured by these methods.

As shown in Table 2 of the main text, the solid residue yield after 650 °C pyrolysis ranges from <1 wt% for clean PE/PP feeds (P1, P3, P4) to ~10 wt% for the most heavily filled/oxidized sample (P5). These solid residues are expected to concentrate inorganic fillers (CaCO_3_), non-volatile metal compounds, carbonaceous char, and char-adsorbed hazardous contaminants that are not volatilized during pyrolysis. Therefore, the NIASs and heteroatom species detected in the volatile/condensable fractions represent only a portion of the total contaminant load in the original waste; the full hazard assessment of the waste streams and pyrolysis process must include characterization of the solid residue phase.

Additionally, the micro-furnace analytical pyrolysis used in this study (0.5–1.0 mg sample in a quartz tube under helium sweep) differs fundamentally from industrial-scale continuous pyrolysis reactors in several respects. First, the extremely small sample mass and thin-film geometry virtually eliminate temperature and barometric gradients within the pyrolysis zone, ensuring near-isothermal, near-instantaneous heating—conditions that are not replicated in industrial screw, fluidized-bed, or rotary-kiln reactors where thick melt layers and variable residence-time distributions prevail. Second, the rapid helium sweep minimizes vapor–solid contact time, suppressing secondary cracking, repolymerization, and resinification reactions that are significant in larger reactors where pyrolysis vapors must traverse a hot coke bed or a long freeboard zone before condensation. Third, mass-transfer limitations in industrial equipment can promote extensive secondary cracking and tar/coke formation, substantially altering the carbon-number distribution, aromatic content, and heteroatom speciation of the condensed oil relative to micro-scale results. Therefore, the pyrolysis product fingerprints reported here represent the intrinsic primary thermal-degradation behavior of the feedstock under idealized analytical conditions, rather than the exact product composition that would be obtained in industrial pyrolysis plants. Scale-up effects—including secondary cracking, tar formation, longer vapor residence times, and contaminant redistribution between gas, liquid, and solid phases—need to be investigated in pilot-scale trials before the present compositional data are used for industrial process design or product-quality prediction. Furthermore, the non-condensable gas fraction (H_2_, CO, CO_2_, CH_4_, C_2_–C_4_ hydrocarbons) produced during pyrolysis at 650 °C is not captured by the GC×GC–TOF–MS configuration used here, because the pyrolysis vapors pass directly into the GC inlet where permanent gases are not retained on the liquid-phase capillary columns employed. Characterization of the gas-phase composition would require a dedicated micro-GC or on-line gas analyzer, which was not available in the present analytical setup. Since polyolefin pyrolysis at 650 °C is accompanied by significant gas formation (typically 5–20 wt% of the feed, depending on polymer type and residence time), the absence of gas-phase data means that the present study provides a detailed fingerprint of the condensable fraction only; the total carbon and energy balance across gas, liquid, and solid phases remains to be established in future work using complementary instrumentation.

## 4. Conclusions

This study combined Py–GC×GC–TOF–MS characterization of pyrolysis oils from six polyolefin-rich waste streams (P1–P6) with headspace GC–MS, solvent-extraction GC–MS, and aqueous migration testing of three recycled polyolefin articles (P7–P9), creating a tiered analytical framework that spans the volatile, condensable, and readily extractable fractions. The key findings and their significance relative to the existing literature are summarized below.

Pyrolysis at 650 °C produced condensable fractions dominated by C5–C30 aliphatic hydrocarbons across all six feeds, but the compositional fingerprint was governed primarily by three feedstock properties: dominant polymer type, pre-oxidation state, and additive burden. Clean PE-rich feeds (P1) yielded n-paraffin/α-olefin-dominated oils with negligible heteroatom species, whereas PP-rich feeds (P3, P4) produced substantially more branched olefins and a modest aromatic fraction (0.09–0.11 area%). The pre-oxidized HDPE samples (P2, P5) retained persistent oxygenated species even at 650 °C, confirming that feedstock oxidation history—not pyrolysis conditions alone—determines the oxygenate burden in the product oil. This finding extends the literature on virgin-polymer pyrolysis [37,38,39,40,41,42], where oxygenated products are rarely reported, by demonstrating that real post-consumer waste introduces oxygen-containing NIASs that survive high-severity thermal treatment. The aromatic content remained below 1 area% for all samples under our fast-pyrolysis conditions (650 °C, 15 s), substantially lower than the 5–15% reported by Jung et al. [40] and Saha et al. [49] under longer residence times, indicating that rapid heating effectively suppresses secondary cyclization and aromatization.

Additive-derived NIASs constituted a small but analytically significant fraction of the pyrolysis oils. The compound 2,4-Di-tert-butylphenol, a recognized degradation product of BHT and Irganox 1010, was detected across multiple samples and confirms—in authentic industrial waste rather than model systems—the additive decomposition pathways quantified by Khan et al. [32] for single-stabilizer pyrolysis. Critically, no phthalate esters, short-chain chlorinated paraffins, or brominated flame-retardant residues were observed in the pyrolysis oils, contrasting sharply with the organochlorine-contaminated products reported by Gao et al. [34] and Strien et al. [35] for PVC-containing mixed-waste feeds. This confirms that NIR-based pre-sorting to high polyolefin purity (>95%) effectively eliminates the halogen-related quality risks that represent the primary obstacle to downstream upgrading and steam-cracker compatibility. Equally significant is the absence of phthalate esters, brominated flame retardants, short-chain chlorinated paraffins, and heavy PAHs from all pyrolysis oils—a negative-finding pattern that reflects the packaging-grade provenance of the feedstocks and distinguishes them from WEEE- or construction-derived waste streams where these substance classes would be expected. This absence profile is directly relevant to regulatory acceptance: it demonstrates that well-sorted polyolefin packaging waste can produce pyrolysis oils free of the most toxicologically concerning legacy contaminant classes, simplifying the downstream purification and compliance pathway under EU PPWR, EFSA, and FDA frameworks.

The complementary VOC/SVOC and aqueous migration analyses of recycled articles (P7–P9) revealed a chemically diverse but mostly low-intensity contaminant profile in which prior-use residues (fragrances, cosmetics, food-related compounds) coexisted with transformed additive chemistry. Importantly, the same antioxidant-degradation NIASs identified in the pyrolysis oils—particularly 2,4-di-tert-butylphenol and the Irganox 1010-derived spiro-dione—also appeared in the SVOC extracts and aqueous migrants of P7–P9, demonstrating that these substances constitute a persistent chemical signature of recycled polyolefins regardless of the processing route. This cross-method convergence is a novel contribution: whereas Horodytska et al. [26] catalogued 134 NIASs in mechanically recycled PE/HDPE and Rung et al. [31] profiled NIASs in post-consumer recyclates, neither study linked the same molecular markers across pyrolysis products and ambient-condition extracts within a single analytical framework. The VOC/SVOC profile also provides insight into the contamination history of the recycled articles. The predominance of cosmetic-, fragrance-, and food-related external contaminants over polymer-degradation products in P7–P9 indicates that these materials were used in household packaging applications where they absorbed ambient chemicals from co-stored consumer products. This contamination signature is distinct from what would be expected for, e.g., agricultural films (where pesticide residues and soil-derived compounds would dominate) or electronic-waste plastics (where BFR degradation products would be prominent). The detection of phthalate esters in P7 and P8—absent from the industrial-grade pyrolyzed feeds P1–P6—further confirms that post-consumer recycled polyolefins acquire a contaminant burden during their use phase that is qualitatively different from the intrinsic additive chemistry of the virgin-polymer, underscoring the need for use-history-aware decontamination protocols rather than one-size-fits-all approaches.

Aqueous migration testing showed that only a subset of the total extractable SVOCs actually migrated into the water simulant: predominantly polar, lower-molecular-weight additive-degradation products and caprolactam (a polyamide cross-contamination marker), while non-polar hydrocarbons and long-chain esters showed negligible transfer. This selectivity pattern has direct regulatory implications: food-contact risk assessment for recycled polyolefins should prioritize polar additive-derived NIAS and cross-contamination markers rather than the total extractable chemical inventory. The detection of caprolactam in all three recycled articles further highlights the value of VOC/SVOC screening as a sorting-quality diagnostic, since polyamide cross-contamination is invisible to FTIR or density-based sorting methods, but is readily flagged by headspace GC–MS.

It must be emphasized that the GC-based methods employed here characterize only the volatile, condensable, and readily extractable fractions, and do not represent the total contaminant load of the source waste. TGA data indicate that solid residue yields range from <1 wt% for clean PE/PP feeds to approximately 10 wt% for the most heavily filled sample (P5), and these residues are expected to concentrate inorganic fillers, non-volatile metals, and char-bound contaminants whose safe management requires separate characterization, including leachability testing and heavy metal screening.

Future work should extend the present framework to polyolefin waste sourced from multiple facilities and geographic regions in order to confirm the transferability of the compositional fingerprints reported here, expand replicate coverage, incorporate residue-phase characterization, and develop quantitative migration data benchmarked against specific regulatory thresholds (EU Regulation 10/2011, EFSA guidance, FDA criteria). A dedicated techno-economic assessment—including life-cycle costing of the tiered analytical workflow, comparison with conventional virgin-polymer production costs, and evaluation of the economic viability of pyrolysis oil upgrading for different feedstock quality tiers—is also recommended as a natural complement to the analytical framework established here, whether undertaken by the present authors or by other research groups. The tiered analytical framework demonstrated here—rapid headspace screening as a first-pass quality gate, followed by comprehensive GC×GC for flagged samples, complemented by targeted migration testing—provides a practical template for linking waste-feed variability to pyrolysis-product quality and for generating the NIAS evidence base that emerging regulatory frameworks for chemical recycling will require.

## Figures and Tables

**Table 1:** Key descriptors of plastic samples P1–P9 used in this study [17].

Sample	Dominant Polymer(s)	Key Feed Descriptors (FTIR, TGA, Source Category)	Analysis Route
P1	LDPE/HDPE blend	PE-rich blend (LDPE + HDPE by FTIR); ash/residue < 1 wt% (TGA); no carbonyl index elevation; negligible inorganic filler. Post-industrial packaging film.	Py–GC×GC–TOF–MS
P2	HDPE	HDPE dominant (FTIR); mildly oxidized (carbonyl band present); CaCO_3_/ash ≈ 6–7 wt% (TGA residue); elevated non-volatile residue relative to clean PE. Post-consumer rigid packaging.	Py–GC×GC–TOF–MS
P3	PP	Isotactic PP (FTIR); ash/residue < 2 wt% (TGA); single-step degradation near 460 °C; no measurable oxidation. Post-industrial injection-molding scrap.	Py–GC×GC–TOF–MS
P4	PP	Isotactic PP (FTIR); ash/residue < 2 wt% (TGA); minimal oxidation or filler signatures. Post-industrial packaging trim.	Py–GC×GC–TOF–MS
P5	HDPE film	HDPE film (FTIR); most strongly oxidized sample (highest carbonyl index); CaCO_3_/ash ≈ 10 wt% (TGA residue); elevated non-volatile residue. Post-consumer agricultural film.	Py–GC×GC–TOF–MS
P6	LDPE/HDPE/PP blend	PE/PP blend (FTIR shows both CH_2_ and CH_3_ signatures); ash/residue ≈ 2–3 wt%; slight oxidation; low inorganic content. Post-consumer mixed-stream sort.	Py–GC×GC–TOF–MS
P7	LDPE/HDPE	PE-rich recycled article (FTIR: LDPE/HDPE); mild oxidation (low carbonyl); low-to-moderate Ca-based filler (≈3–5 wt% ash). Post-consumer household packaging.	VOC/SVOC/migration
P8	HDPE	HDPE-rich recycled article (FTIR); mild oxidation; moderate Ca-based filler (≈5–7 wt% ash). Post-consumer rigid container.	VOC/SVOC/migration
P9	HDPE/PP mix	Mixed HDPE/PP article (FTIR shows both PE and PP bands); slight unsaturation/oxidation; low inorganic content (<2 wt% ash). Post-consumer mixed packaging.	VOC/SVOC/migration

**Table 2:** Mass balance before and after pyrolysis of plastic waste samples P1–P6 at 450 °C and 650 °C.

Name	Mass (mg) Before Pyrolysis	Mass (mg) After Pyrolysis	Temperature (°C)
P1_450	5.517	5.544	450
P2_450	4.582	4.611	450
P3_450	4.27	4.36	450
P4_450	4.029	4.027	450
P5_450	3.992	4.032	450
P6_450	4.464	4.495	450
P1_650	2.611	1.452	650
P2_650	2.085	0.274	650
P3_650	2.577	0.477	650
P4_650	2.409	0.395	650
P5_650	2.083	0.596	650
P6_650	2.121	0.389	650

**Table 3:** Semi-volatile compounds (>C_7_) identified by GC×GC–TOF–MS in the condensates obtained from each polymer stream (P1–P6) at 650 °C. Only molecules present at ≥2% of the total chromatographic area in at least one sample are listed; blank cells indicate <2% or not detected.

Compound	P1	P2	P3	P4	P5	P6
**Alcohols**						
1-Decanol, 2-hexyl-	35.63	13.22	2.5	4.56	17.14	
1-Eicosanol	5.61	2.1			13.42	7.92
n-Nonadecanol-1	3.16	3.37				
2-Hexyl-1-octanol	2.75	3.16			5.12	
2-Ethyl-1-dodecanol	2.24					
11-Dodecen-1-ol, 2,4,6-trimethyl-,			2.36	2.85		
11-Hexadecen-1-ol, (Z)-					2.36	
1-Octanol, 2-butyl-			2.16	2.37		
2-Isopropyl-5-methyl-1-heptanol			6.08	7.9		3.76
n-Heptadecanol-1		2.54				
Tricosan-2-ol			12.12	13.97		
**Olefins**						
1-Docosene	9.04	5.45				4.76
1,19-Eicosadiene	4.27	9.31			9.44	2.99
1-Tetracosene	2.95	3.13	2.19	11	2.23	
1-Nonadecene	2.85	3.77			8.77	2.47
1-Heptadecene						4.21
1-Hexacosene		8.77				3.25
2,4-Dimethyl-1-heptene						2.36
Cetene					2.89	
Nonacos-1-ene			16.65	18.67		10.47
Pentacos-1-ene			3.3			
**Esters**						
Oxalic acid, allyl octadecyl ester	2.4		11.92	3.11		9
Carbonic acid, eicosyl vinyl ester		9.15			9.03	8.1
Oxalic acid, allyl hexadecyl ester						4.95
Oxalic acid, allyl tridecyl ester			6.07			
**Alkenal**						
cis-4-Decenal		2.89				
E-15-Heptadecenal					3.34	
**Cycloparaffins**						
Cyclotetradecane, 1,7,11-trimethyl-4-(1-methylethyl)-			15.78	10.8		
**Paraffins**						
Nonadecane		3.05			2.78	
**Alkyne**						
1-Octadecyne	4.19				4.72	

**Table 4:** The hydrocarbon composition of the pyrolysis products measured using Py–GC×GC–MS.

Category	Concentration (Area %)					
Category	P1	P2	P3	P4	P5	P6
Alcohol	53.52	23.92	20.59	22.12	17.75	32.09
α-olefines	25.5	46.34	26.49	25.8	35.82	34.08
Diolefins	4.57	8	1.11	1.31	4.53	2.54
Iso-olefins	1.51	1.83	5.75	3.73	3.03	6.07
Naphthenes	0.27	0.94	21.56	27.29	20.34	8.8
n-paraffins	1.8	8.67			2.64	
Iso-paraffins				0.19		0.21
Ester	5.59	4.79	14.47	10.7	4.28	10.27
Alkyne	4.31	1.34	2.9	1.81	0.72	0.64
Oxirane	2.73	1.08	0.68	1.8	5.61	0.99
Aromatics		0.06	0.11	0.09	0.45	0.85
Si		2.45			0.11	
Cyclic alcohols			5.7	3.03	0.47	1
Ether			0.34	0.99	2.25	2.36

**Table 5:** Static headspace VOCs (≤C_14_) detected by GC–MS in samples P7–P9 after incubation at 80 °C for 1 h. Compounds are grouped by chemical family; peak area intensity is coded as 1 = low, 2 = medium, 3 = high. Designations: A = additive intentionally present in the original polymer, AD = additive-degradation product, PD = polymer-degradation product, C = external contaminant, deg = secondary degradation.

Compound Name	Peak Intensity			Classification		
Compound Name	P7	P8	P9	Type	Possible Origin	Odorants
**Hydrocarbons**						
10-Heneicosene (c,t)	1			PD	Polymer degradation	
1-Dodecene	1			PD	Polymer degradation	
1-Nonadecene			2	PD	Polymer degradation	
1-Tetradecene	1		3	PD	Polymer degradation	
2,4-Dimethyl-1-heptene	1	2	1	C	Polymer degradation	Strong pungent plastic odor
3-Dpdecene			1	PD	Polymer degradation	
3-Eicosene, (E)-		1		PD	Polymer degradation	
Cetene	1		3	PD	Polymer degradation	
Decane	1		1	PD	Polymer degradation	
Decane, 4-methyl-		1		PD	Polymer degradation	
Dodecane	1	3		PD	Polymer degradation	
Dodecane, 2,7,10-trimethyl-		3		PD	Polymer degradation	
Eicosane, 2-methyl-		1		PD	Polymer degradation	
Heptacosane		1		PD	Polymer degradation	
Heptadecane, 2,6,10,15-tetramethyl-		1		AD	Lubricant	
Heptadecane, 4-methyl-		1		PD	Polymer degradation	
Heptane, 2,3-dimethyl-		1		C	Processing aid	
Heptane, 2,4-dimethyl-		3		C	Processing aid	
Heptane, 4-methyl-		1		PD	Polymer degradation	
Hexadecane	1		1	PD	Polymer degradation	
Hexadecane, 2,6,11,15-tetramethyl-		1		AD	Lubricant	
Hexane, 2,3,5-trimethyl-		1		C	Processing aid	
Nonadecane	2	1		PD	Polymer degradation	
Octadecane, 2-methyl-	1	1	1	PD	Processing aid	
Octane	1			PD	Polymer degradation	
Octane, 4-methyl-		2		PD	Polymer degradation	
Tetradecane	2	1	2	PD	Polymer degradation	
Tridecane	1			PD	Polymer degradation	
Undecane	1	3		PD	Polymer degradation	
Undecane, 4-methyl-		1		PD	Polymer degradation	
α-Methylstyrene	3	2	2	PD	Polymer degradation	
Tetradecane, 4-methyl-		1		PD	Polymer degradation	
**Alcohols**						
1-Decanol, 2-hexyl-		1		C	Daily chemical products-related	
1-Butanol		1		C	Cosmetics	
1-Octanol, 2,7-dimethyl-		2		C	Cosmetics/Industrial Solvent	Camphor-like odor
1-Octanol, 2-butyl-		1		C	Daily chemical products related	
2,2-dimethylphenylmethanol	1	1		C	Daily chemical products-related	
2-Hexyl-1-octanol			1	C	Flavor and fragrance agent	
2-Undecanethiol, 2-methyl-		1		C	Daily chemical products-related	Pungent and garlic-like odor
Cyclobutanol	1	1	1	C	Daily chemical products-related	
**Aldehydes and Ketones**						
2-Pentenal, 2-methyl-		1		C	Flavor and fragrance agent	
Decanal		1		PD or C	Cosmetics	
Heptanal		1		PD or C	Flavor and fragrance agent	
Hexanal		1		PD or C	Flavor and fragrance agent	
Nonanal	1			PD or C	Cosmetics	
Octanal		1		PD or C	Cosmetics	
Pentanal	1	1		PD or C	Flavor and fragrance agent	
2-Heptanone, 4,6-dimethyl-		1		C	Daily chemical products-related	
2-Heptanone, 4-methyl-		1		C	Flavor and fragrance agent	
Acetophenone	1	1	1	C	Daily chemical products-related	
Cyclopentanone, 2-(1-methylpropyl)-		1		C	Daily chemical products-related	
**Others**						
1-Propanol, 2-amino-, (±)-	1			C	Industrial solvent/Adhesive	
2,3-Epoxybutane	1	1		C	Pharmaceutical-related	
2,6-Di-tert-butylbenzoquinone		1		AD	Oxidation phosphite additives	
2,6-di-tert-butyl-4-ethylphenol	1			AD	Antioxidant	
2-Aminononadecane	1	1	1	C	Lubricant additive	Mild amine odor
2-Bromo dodecane		1		AD	Plasticizer intermediate	
2-Butanamine, 3-methyl-			1	C	Pharmaceutical-related	Strong fishy odor
2-Ethylacrolein		1		C	Processing aid	Pungent and acrid odor
2-Octanamine			1	C	Daily chemical products related	
2-Pentanamine, 4-methyl-	1		1	C	Daily chemical products-related	
3-(Prop-2-enoyloxy)dodecane	1			C	Flavor and fragrance agent	
3,3-Dimethyl-4-methylamino-butan-2-one	1		1	C	Daily chemical products-related	
3,5-di-tert-Butyl-4-hydroxybenzaldehyde	1			AD	Irganox 1076 oligomer	
Benzene, (1-methoxy-1-methylethyl)-	1			C	Daily chemical products-related	
Benzene, 1,3-bis(1,1-dimethylethyl)-	1	1	1	C	Processing aid	
Benzene, 1,3-bis(1-methylethenyl)-	1			C	Processing aid	
Benzene, 1-ethynyl-4-methyl-		1		C	Processing aid	
Butanal, 3-hydroxy-		1		C	Daily chemical products-related	
Caprolactam	2			PD	Processing aid	
Cis-bicyclo [4.2.0]octane		1	1	C	Processing aid	
Cyclotrisiloxane, hexamethyl-	1		1	C	Cosmetics/Lubricant	
Ethanone, 1-(2,3-dihydro-1H-inden-5-yl)-	1			C	Daily chemical products-related	Pungent odor
Ethyne, fluoro-			1	C	Processing aid	
Furan, 2,5-dihydro-2,5-dimethyl-	-	1		C	Processing aid	
Hexacosyl acetate		1		C	Flavor and fragrance agent	
Hydroxylamine, O-decyl		1	1	C	Processing aid	Ammonia-like odor
N-dl-Alanylglycine	1			C	Pharmaceutical/Food related	
n-Hexylmethylamine	1		1	C	Processing aid	
Pentanamide				C	Pharmaceutical-related	
Phenol, 2-(3-hydroxy-3-methyl-1-butenyl)-, (Z)-	1			C	Daily chemical products-related	
Phenol, 2,4-bis(1,1-dimethylethyl)-	1		1	AD	Antioxidant	
Propanamide				C	Pharmaceutical-related	
R-(-)-Cyclohexylethylamine	1	1	1	C	Processing aid	Strong ammonia-like odor
sec-Butylamine	1			C	Processing aid	

**Table 6:** Semi-volatile organic compounds (SVOCs) identified by GC–MS in hexane extracts of samples P7–P9. Compounds are grouped by chemical family; only those contributing ≥1% of the SVOC total-ion area in at least one sample are listed. Peak-area intensity: 1 = low, 2 = medium, 3 = high. Source codes: A = intentional additive, AD = additive-degradation product, PD = polymer-degradation product, C = external contaminant, deg = secondary degradation.

Compound Name	Peak Intensity			Classification	
Compound Name	P7	P8	P9	Type	Possible Origin
**Hydrocarbons**					
Cetene	1	2		PD	
Decane, 4-methyl-			1	PD	
Dodecane			2	PD	
Dodecane, 2,6,10-trimethyl-			1	C	Cosmetic-related
Dodecane, 2,6,11-trimethyl-			2	C	Cosmetic-related
Eicosane	2			PD	
Heptacosane	3		2	PD	
Heptadecane, 2,6,10,15-tertamethyl-			1	C	Processing aid
Hexadecane		1	1	PD	
Hexadecane, 2,6,10,14-tetramethyl-			1	C	Processing aid
Octadecane	1		1	PD	
Octadecane, 3-ethyl-5-(2-ethylbutyl)-	1			C	Processing aid
Octadecane, 6-methyl-		1	1	PD	
Octane, 4-methyl-			1	PD	
Pentadecane			2	PD	
Pentadecane, 3-methyl-	1			PD	
Tetradecane	1		1	PD	
Tetradecane, 2,6,10-trimethyl-	1		2	C	Cosmetic- or pharmaceutical-related
Undecane			2	PD	
Undecane, 4-methyl-			2	PD	
1-Nonadecene	2	3		PD	
1-Octadecene	3			PD	
1-Pentadecene,2-methyl-		1		PD	
17-Pentatriacontene	1	2	1	AD	Lubricant
**Esters**					
1-Propene-1,2,3-tricarboxylic acid, tributyl ester	3	2	1	AD	Plasticizer
9-Octadecenoic acid (Z)-, tetradecyl ester		1		C	-
Decanedioic acid, bis(2,2,6,6- tetramethyl-4-piperidinyl) ester			2	C	Pharmaceutical-related
Hexadecanoic acid, 1-(hydroxymethyl) -1,2-ethanediyl ester		1		C	Cosmetic-related or lubricant
Hexadecanoic acid, butyl ester	1	1		C	Daily chemical products-related
Isopropyl myristate			1	C	Cosmetic-related
Octadecanoic acid, 2-hydroxy-1,3-propanediyl ester	3	1		C	Cosmetic-related
Octadecanoic acid, 4-hydroxy-, methyl ester		1		C	Daily chemical products-related
Octadecanoic acid, butyl ester	2	2		C	Daily chemical products-related
Oleic acid, 3-(octadecyloxy)propyl ester	2	1		C	Cosmetic-related
Oleic acid, eicosyl ester	1	1		C	Cosmetic-related
Oxalic acid, ethyl 2-isopropylphenyl ester	2			AD	Plasticizer
Trichloroacetic acid, pentadecyl ester			1	A	Plasticizer
**Alcohols**					
1,4:3,6-Dianhydro-α-d-glucopyranose			1	C	Food-related
1-Dodecanol, 3,7,11-trimethyl-			1	C	Daily chemical products-related
1-Eicosanol	2			C	Daily chemical products-related
1-Hexadecanol, 2-methyl-	1	2	1	C	Daily chemical products-related
1-Octanol, 2,2-dimethyl-			1	AD	Plasticizer
2,2,6,6-Tetramethyl-4-piperidinol	1			C	Pharmaceutical-related
2-Hexadecanol		1		AD	Lubricant
2-Methyl-E,E-3,13-Octadecadien-1-ol	1			C	Pharmaceutical-related
3-Hexadecanol			1	C	Cosmetic-related or surfactant
3-Isopropyl-6,7-dimethyltricyclo [4.4.0.0(2,8)] decane-9,10-diol		1		AD	Plasticizer or flame retardant
Behenic alcohol	2	1		C	Cosmetic-related or lubricant
Ethanol, 2-(octadecyloxy)-	1	2		C	Cosmetic-related or lubricant
Ethyl iso-allocholate	1	1	1	C	Pharmaceutical-related
n-Tetracosanol-1	2	2		C	Pharmaceutical-related
Octacosanol	1	2		C	Pharmaceutical-related
tert-Hexadecanethiol	1	1		C	Processing aid
α-N-Normethadol	1			C	Pharmaceutical-related
**Benzene derivatives**					
1,3-Benzenedicarboxylic acid, bis(2-ethylhexyl) ester	1			A	Plasticizer
1,3-Diacetylbenzene	1			C	Pharmaceutical-related
1,4-Benzenediol, 2,6-bis(1,1-dimethylethyl)-		1		A	Antioxidant
3,5-di-tert-Butyl-4-hydroxyacetophenone		1	1	A	Antioxidant and stabilizer
4[h]-Pyridone, 1-benzyl-3,5-dichloro-2,6-dimethyl-			1	C	Pesticide
Benzene, 1,1′-(1,1,2,2-tetramethyl-1,2-ethanediyl)bis-	2			C	Processing aid
Benzene, 1,3-bis(1,1-dimethylethyl)-			1	AD	Antioxidant
Benzeneethanol, α-methyl-3-(1-methylethyl)-	1			C	Daily chemical products-related
Benzenepropanoic acid, 3,5-bis(1,1-dimethyl ethyl)-4-hydroxy-, octadecyl ester		3		C	Pharmaceutical-related
Benzenmethanol, a,a-dimethyl-	1			C	Processing aid
Benzestrol	1			C	Pharmaceutical-related
Diisooctyl phthalate	1	2		A	Plasticizer
Phenol, 2,4-bis(1,1-dimethylethyl)-	1	2	1	AD	Antioxidant
Phenol, 2,6-bis(1,1-dimethylethyl)-4-ethyl-	1			AD	Antioxidant
Phenol, 2,6-di-tert-butyl-4-ethyl-	1			AD	Antioxidant
Phenol, 2-methyl-4-(1,1,3,3-tetramethylbutyl)-	2			AD	Antioxidant
Phenol, 4-(1,1,3,3-tetramethylbutyl)-	1			AD	Antioxidant or stabilizer
Phenol, 4-(1,1-dimethylpropyl)-	1			C	Pharmaceutical-related
Phthalic acid, butyl tetradecyl ester	1			A	Plasticizer
p-Octylacetophenone		1		C	Cosmetic-related
γ-Chlorobutyrophenone			1	C	Surfactant
**Acids, Aldehydes, Amides, Ketones**					
Octadecanoic acid		2		C	Daily chemical products-related
cis-13-Eicosenoic acid	1			C	Food-related
n-Hexadecanoic acid	1		1	C	Daily chemical products-related
Pterin-6-carboxylic acid			1	C	Pharmaceutical-related
10-Octadecenal			1	C	Daily chemical products-related
5-Octadecenal		1	1	C	Daily chemical products-related
8-Octadecenal		1		C	Daily chemical products-related
E-15-Heptadecenal		3		C	Food-related
13-Docosenamide, (Z)-	1		3	C	Daily chemical products-related
Cis-11-Eicosenamide			1	C	Lubricant
2,2,7,7Tetramethyltricyclo [6.2.1.0(1,6)] undec-4-en-3-one		1		C	Pharmaceutical-related
2-Pentanone, 4-hydroxy-4-methyl-			2	C	-
7,9-Di-tert-butyl-1-oxaspiro(4,5)deca-6,9-diene-2,8-dione	1	2	1	AD	Antioxidant degradation product
**Others**					
2-Bromo dodecane			1	AD	Plasticizer
2-Trifluoroacetoxytridecane			1	A	Plasticizer
Caprolactam	3		1	PD	Polyamide contaminant/degradation product
Octadecanal, 2-bromo-	1	1	1	AD	Plasticizer or lubricant
Octasiloxane, 1,1,3,3,5,5,7,7,9,9,11, 11,13,13,15,15-hexadecamethyl-		1		A	Lubricant
Octatriacontyl pentafluoropropionate		1	1	C	Surfactant or lubricant, toxic
Silane, diethylheptyloxyoctadecyloxy-	1	3	2		Processing aid
Triallyl isocyanurate	1			AD	Flame retardant or plasticizer
Tributyl acetylcitrate	1		3	A	Plasticizer

**Table 7:** Organic migrants detected in aqueous food simulant (10 d/40 °C) from samples P7–P9. Only compounds with a GC–MS peak intensity ≥ 1 are listed (1 = trace, 2 = moderate, 3 = dominant).

Compound Name	Peak Intensity			Possible Origin
Compound Name	P7	P8	P9	
**Benzene derivatives**				
1,2-Dimethoxy-4-(1-methoxy-1-propenyl)benzene			1	Pharmaceutical-related
2,4-Dimethyl-5,6-dimethoxy-8-aminoquinoline	1			Pharmaceutical-related
3,5-di-tert-Butyl-4-hydroxycinnamic acid			2	Antioxidant and stabilizer
Benzenesulfonamide, N-ethyl-2-methyl-			1	Sweetener in food industry
Benzyl alcohol, α,α-dimethyl-p-isopropyl-	2			Cosmetic- or Pharmaceutical-related
Ethanone, 1,1′-(1,4-phenylene)bis-	1			Plasticizer or UV absorber
Phenol, 2,6-bis(1,1-dimethylethyl)-		1	1	Antioxidant
p-Octylacetophenone		2	2	Food industry
**Acids, Alcohols, Esters, Ketones**				
Dimethylmuconic acid			1	Pharmaceutical-related
1-(3-Methoxymethyl-2,4,6-trimethylphenyl)ethanol	1			
1,4:3,6-Dianhydro-α-d-glucopyranose			2	Food-related
Dibutyl itaconate			1	Plasticizer
Furan, 2-[(2-ethoxy-3,4-dimethyl-2-cyclohexen-1-ylidene)methyl]-	1			Cosmetic- or food-related
2,5-Cyclohexadiene-1,4-dione, 2,6-bis (1,1-dimethylethyl)-		1	1	Pharmaceutical-related
2,5-di-tert-Butyl-1,4-benzoquinone		1	1	Stabilizer
7,9-Di-tert-butyl-1-oxaspiro(4,5)deca-6,9- diene-2,8-dione		3	3	Antioxidant degradation product
**Others**				
4-Piperidinol, 2,2,6,6-tetramethyl-	1		3	Processing aid
Caprolactam	3	2	2	Polyamide contaminant/degradation product
Cyclobarbital			1	Pharmaceutical-related
Hexasiloxane, 1,1,3,3,5,5,7,7,9,9,11,11-dodecamethyl-	1	1	1	Plasticizer
l-Guanidinosuccinimide	1			Pharmaceutical-related
Morpholinomethyl urea i			1	Corrosion inhibitor or flame retardant
Octasiloxane, 1,1,3,3,5,5,7,7,9,9,11,11,13,13,15,15-hexadecamethyl-	1	1	1	Plasticizer or lubricant
Pregn-5-ene-3,11,20-trione, cyclic 3,20-bis (1,2-ethanediyl acetal)			1	Pharmaceutical-related

**Table 8:** Screening-based implications of integrated analytical observations for samples P1–P9.

Sample	Main Analytical Flags	Main Implication	Indicative Routing/Pretreatment
**P1**	PE-rich stream; negligible ash/filler; no clear oxidation; hydrocarbon-dominant pyrolysis fingerprint with minimal oxygenates	Relatively straightforward polyolefin stream	Mechanical recycling is favored where product-quality specifications are met; chemical recycling is also technically feasible
**P2**	Mildly oxidized HDPE; CaCO_3_/ash ~6–7 wt%; low-level oxygenated species in pyrolysis products	Oxidation and filler burden may reduce product quality relative to cleaner PE streams	Pretreatment and/or downstream upgrading may be more important than for cleaner PE-rich feeds
**P3**	Clean PP; low inorganic burden; branched hydrocarbon-rich pyrolysis fingerprint	Low-contaminant PP-rich stream	Strong candidate for mechanical recycling; chemical recycling also remains feasible
**P4**	Clean PP; minimal oxidation/filler signatures; product fingerprint similar to P3	Low-contaminant PP-rich stream	Strong candidate for mechanical recycling; chemical recycling also remains feasible
**P5**	Most strongly oxidized PE sample; CaCO_3_/ash ~10 wt%; persistent low-level oxygenated NIASs	Highest upgrading burden among the pyrolyzed samples	Stronger candidate for chemical recycling or lower-sensitivity applications; downstream upgrading is likely to be more important
**P6**	Mixed PE/PP feed; slight oxidation; low inorganic content; hybrid pyrolysis fingerprint	Mixed-polymer character may complicate closed-loop mechanical recycling	Chemical recycling may provide a more robust routing option than direct closed-loop reuse
**P7**	PE-rich recycled article; caprolactam at high intensity (nylon-6 cross-contamination marker); diverse VOC/SVOC/migrant profile	Prior-use contamination signatures remain relevant	Reuse decisions should be application-specific and supported by decontamination and screening
**P8**	PE-rich recycled article; moderate filler burden; antioxidant-degradation signatures in extractable/migrating fractions	Additive-related residues remain relevant for end use	Non-sensitive reuse may be feasible after QA/QC; sensitive uses require additional scrutiny
**P9**	Mixed HDPE/PP article; low inorganic content; cross-contamination markers such as caprolactam	Mixed-polymer behavior and NIAS markers remain relevant	Final routing should depend on separation feasibility, intended use, and additional quality screening

## Data Availability

The original contributions presented in this study are included in the article/Supplementary Materials. Further inquiries can be directed to the corresponding author.

## Supplementary Materials

The following supporting information can be downloaded at: https://www.mdpi.com/article/10.3390/polym18111381/s1, The Supplementary Information includes Figures S1–S6 (450 °C GC×GC–TOF–MS screening maps of P1–P6), Figure S7 (representative pre-pyrolysis FTIR spectra of P1–P9 with polymer references), and Figure S8 (TGA/DTG profiles of P1–P9 used to justify the 450 °C versus 650 °C analytical pyrolysis comparison).

## Nomenclature

FT-IR	Fourier Transform Infrared Spectroscopy
DSC	Differential Scanning Calorimetry
TGA	Thermal Gravimetric Analysis
CCD	Charge-Coupled Device
NIAS	Non-Intentionally Added Substances
LDPE	Low-Density Polyethylene
HDPE	High-Density Polyethylene
PP	Polypropylene
PVC	Polyvinyl Chloride
UV	Ultraviolet
PBDEs	Polybrominated Diphenyl Ethers
PFAS	Perfluoroalkyl Substances
POPs	Persistent Organic Pollutants
SCCPs	Short-Chain Chlorinated Paraffins
PCBs	Polychlorinated Biphenyls
REACH	Registration, Evaluation, Authorization, and Restriction of Chemicals
UNEP	United Nations Environment Programme
Py–GC×GC–TOF–MS	Pyrolysis coupled with Comprehensive Two-Dimensional Gas Chromatography Time-of-Flight Mass Spectrometry
GC	Gas Chromatography
TOF-MS	Time-of-Flight Mass Spectrometry
GC×GC	Comprehensive Two-Dimensional Gas Chromatography
CDS	Chemical Delivery Systems (specific to the Pyroprobe 6150 model)
HP-5MS UI	High-Performance 5% Phenyl Methylpolysiloxane Ultra Inert
BPX-50	Biphenyl Polysilphenylenesiloxane (mid-polar column)
EI+	Electron Ionization (Positive Mode)
NIST	National Institute of Standards and Technology
EPA	Environmental Protection Agency
NIH	National Institutes of Health
m/z	Mass-to-Charge Ratio
Hz	Hertz
GCImageTM	Gas Chromatography Image (software)
eV	Electron Volt

## References

[1] United States Food and Drug Administration (FDA) (2023). Chemistry Guidance for Recycled Plastics in Food Packaging. FDA Guidance Document.

[2] Saxena S. (2024). Pyrolysis and Beyond: Sustainable Valorization of Plastic Waste. Appl. Energy Combust. Sci..

[3] Almroth B.C. (2023). Scientists found hundreds of toxic chemicals in recycled plastics. ScienceDaily. https://www.sciencedaily.com/releases/2023/11/231110112511.htm.

[4] Almroth B.C., Carmona E., Chukwuone N., Dey T., Slunge D., Backhaus T., Karlsson T. (2025). Addressing the toxic chemicals problem in plastics recycling. Camb. Prism. Plast..

[5] Geueke B., Groh K., Muncke J. (2018). Food packaging in the circular economy: Overview of chemical safety aspects for commonly used materials. J. Clean. Prod..

[6] Geueke B., Phelps D.W., Parkinson L.V., Muncke J. (2023). Hazardous chemicals in recycled and reusable plastic food packaging. Camb. Prism. Plast..

[7] Groh K.J., Backhaus T., Carney-Almroth B., Geueke B., Inostroza P.A., Lennquist A., Leslie H.A., Maffini M., Slunge D., Trasande L. (2019). Overview of known plastic packaging-associated chemicals and their hazards. Sci. Total Environ..

[8] Groh K.J., Geueke B., Martin O., Maffini M., Muncke J. (2021). Overview of intentionally used food contact chemicals and their hazards. Environ. Int..

[9] Hahladakis J.N., Velis C.A., Weber R., Iacovidou E., Purnell P. (2018). An overview of chemical additives present in plastics: Migration, release, fate and environmental impact during their use, disposal and recycling. J. Hazard. Mater..

[10] Jones K.C. (2021). Persistent organic pollutants (POPs) and related chemicals in the global environment: Some personal reflections. Environ. Sci. Technol..

[11] Weber R., Ashta N.M., Aurisano N., Wang Z., Outters M., De Miguel K., Schlummer M., Blepp M., Wiesinger H., Andrade H. (2023). Chemicals in Plastics—A Technical Report.

[12] Spring M., Schröder P., Popovici A., O’Meara N., Corsi I., Aliani S., Boodhoo K., Gobin J., Godoy-Faúndez A., Kahru A. (2025). Effective progress and implementation of the INC-5 plastics treaty through scientific guidance. Nat. Sustain..

[13] Aanesen M., Ahi J.C., Abate T.G., Khan F.R., de Vries F.P., Kite-Powell H., Beaumont N.J. (2024). Insights from international environmental legislation and protocols for the global plastic treaty. Sci. Rep..

[14] Farrelly T., Brander S., Thompson R., Almroth B.C. (2025). Independent science key to breaking stalemates in global plastics treaty negotiations. Camb. Prism. Plast..

[15] Farrelly T., Gammage T., Carney Almroth B., Thompson R. (2024). Global plastics treaty needs trusted science. Science.

[16] Vince J., Carney Almroth B., De Miranda Grilli N., Dwivedi V., Stöfen-O’Brien A., Beyer J. (2024). The Zero Draft Plastics Treaty: Gaps and challenges. Camb. Prism. Plast..

[17] Chen A., Saxena S., Samaras V.G., Dally B. (2026). A Tiered Multi-Technique Decision-Support Framework for Contaminant Screening and Recycling-Route Assignment of Mixed Plastic Waste. Polymers.

[18] European Commission (2025). Regulation (EU) 2025/40 of the European Parliament and of the Council on packaging and packaging waste. Off. J. Eur. Union.

[19] European Food Safety Authority (EFSA) (2021). Note for Guidance For the Preparation of an Application for the Safety Assessment of a Substance to be used in Plastic Food Contact Materials. EFSA J..

[20] Hochegger A., Gollner T., Gadermaier B., Gruber S., Baumgartner S., Malachova A. (2023). One-dimensional and comprehensive two-dimensional gas chromatographic approaches for the characterization of post-consumer recycled plastic materials. Anal. Bioanal. Chem..

[21] Toraman H.E., Dijkmans T., Djokic M.R., Van Geem K.M., Marin G.B. (2014). Detailed compositional characterization of plastic waste pyrolysis oil by comprehensive two-dimensional gas-chromatography coupled to multiple detectors. J. Chromatogr. A.

[22] Ong H.-T., Samsudin H., Soto-Valdez H. (2022). Migration of endocrine-disrupting chemicals into food from plastic packaging materials: An overview of chemical risk assessment, techniques to monitor migration, and international regulations. Crit. Rev. Food Sci. Nutr..

[23] Radhakrishnan H., Mohammed A.A.B.A., Coffman I., Bai X. (2025). Influence of Functional Additives, Fillers, and Pigments on Thermal and Catalytic Pyrolysis of Polyethylene for Waste Plastics Upcycling. Green Chem..

[24] Staub C. Study: E-Plastics Additives Present in Home Goods. Plast. Recycl. Update 2024. https://resource-recycling.com/plastics/2024/12/04/study-e-plastics-additives-present-in-home-goods/.

[25] Wiesinger H., Wang Z., Hellweg S. (2021). Deep dive into plastic monomers, additives, and processing aids. Environ. Sci. Technol..

[26] Horodytska O., Cabanes A., Fullana A. (2020). Non-intentionally added substances (NIAS) in recycled plastics. Chemosphere.

[27] Beccaria M., Piparo M., Zou Y., Stefanuto P.-H., Purcaro G., Mendes Siqueira A.L., Maniquet A., Giusti P., Focant J.-F. (2023). Analysis of mixed plastic pyrolysis oil by comprehensive two-dimensional gas chromatography coupled with low- and high-resolution time-of-flight mass spectrometry with the support of soft ionization. Talanta.

[28] Dao Thi H., Djokic M.R., Van Geem K.M. (2021). Detailed group-type characterization of plastic-waste pyrolysis oils: By comprehensive two-dimensional gas chromatography including linear, branched, and di-olefins. Separations.

[29] Kusenberg M., Roosen M., Zayoud A., Djokic M.R., Dao Thi H., De Meester S., Ragaert K., Kresovic U., Van Geem K.M. (2022). Assessing the feasibility of chemical recycling via steam cracking of untreated plastic waste pyrolysis oils: Feedstock impurities, product yields and coke formation. Waste Manag..

[30] Wu S., Wu X., Li H., Li D., Zheng J., Lin Q.-B., Nerin C., Zhong H., Dong B. (2022). The characterization and influence factors of semi-volatile compounds from mechanically recycled polyethylene terephthalate (rPET) by combining GC × GC-TOFMS and chemometrics. J. Hazard. Mater..

[31] Rung C., Welle F., Gruner A., Springer A., Steinmetz Z., Munoz K. (2023). Identification and Evaluation of (Non-) Intentionally Added Substances in Post-Consumer Recyclates and Their Toxicological Classification. Recycling.

[32] Khan R., Perez B.A., Toraman H.E. (2024). Comparative analysis of additive decomposition using one-dimensional and two-dimensional GC with FID/TOF-MS. J. Anal. Appl. Pyrolysis.

[33] Turner A., Filella M. (2021). Hazardous metal additives in plastics and their environmental impacts. Environ. Int..

[34] Gao P., Hu Z., Sheng Y., Pan W., Ding L., Tang L., Chen X., Wang F. (2023). Pyrolysis of municipal plastic waste: Chlorine distribution and formation of organic chlorinated compounds. J. Hazard. Mater..

[35] Strien J.R.J., Genuino H.C., van Eijk M.C.P., Deuss P.J., Heeres H.J. (2023). Pyrolysis of Polyolefin-Enriched Mixed Plastic Waste Streams: Effects of Pretreatments and Presence of PVC. Energy Fuels.

[36] Xayachak T., Haque N., Parthasarathy R., King S., Emami N., Lau D., Pramanik B.K. (2022). Pyrolysis for plastic waste management: An engineering perspective. J. Environ. Chem. Eng..

[37] Scheirs J., Kaminsky W. (2006). Feedstock Recycling and Pyrolysis of Waste Plastics: Converting Waste Plastics into Diesel and Other Fuels.

[38] Lopez G., Artetxe M., Amutio M., Bilbao J., Olazar M. (2017). Thermochemical routes for the valorization of waste polyolefinic plastics to produce fuels and chemicals. A review. Renew. Sustain. Energy Rev..

[39] Mastral F.J., Esperanza E., García P., Juste M. (2002). Pyrolysis of high-density polyethylene in a fluidised bed reactor. Influence of the temperature and residence time. J. Anal. Appl. Pyrolysis.

[40] Jung S.-H., Cho M.-H., Kang B.-S., Kim J.-S. (2010). Pyrolysis of a fraction of waste polypropylene and polyethylene for the recovery of BTX aromatics using a fluidized bed reactor. Fuel Process. Technol..

[41] Ahmad I., Khan M.I., Khan H., Ishaq M., Tariq R., Gul K., Ahmad W. (2015). Pyrolysis Study of Polypropylene and Polyethylene Into Premium Oil Products. Int. J. Green. Energy.

[42] Onwudili J.A., Insura N., Williams P.T. (2009). Composition of products from the pyrolysis of polyethylene and polystyrene in a closed batch reactor: Effects of temperature and residence time. J. Anal. Appl. Pyrolysis.

[43] Akoueson F., Chbib C., Monchy S., Paul-Pont I., Doyen P., Dehaut A., Duflos G. (2021). Identification and quantification of plastic additives using pyrolysis-GC/MS: A review. Sci. Total Environ..

[44] Chen Y., Li H., Huang H., Zhang B., Ye Z., Yu X., Shentu X. (2023). Recent advances in non-targeted screening of compounds in plastic-based/paper-based food contact materials. Foods.

[45] He Y.-J., Qin Y., Zhang T.-L., Zhu Y.-Y., Wang Z.-J., Zhou Z.-S., Xie T.-Z., Luo X.-D. (2023). Migration of (non-) intentionally added substances and microplastics from microwavable plastic food containers. J. Hazard. Mater..

[46] Ureel Y., Chacón-Patiño M.L., Kusenberg M., Ginzburg A., Rodgers R.P., Sabbe M.K., Van Geem K.M. Detailed Characterization of Plastic Pyrolysis Oils and Their Contaminants by FT-ICR MS and GC × GC. Proceedings of the 2024 Spring Meeting and 20th Global Congress on Process Safety.

[47] Cuthbertson A.A., Lincoln C., Miscall J., Stanley L.M., Maurya A.K., Asundi A.S., Tassone C.J., Rorrer N.A., Beckham G.T. (2022). Characterization of polymer properties and identification of additives in commercially available bio-based and conventional food packaging materials. Polym. Test..

[48] Zou L., Xu R., Wang H., Wang Z., Sun Y., Li M. (2024). Chemical recycling of polyolefins: A closed-loop cycle of waste to olefins. Natl. Sci. Rev..

[49] Saha B., Vedachalam S., Dalai A.K., Saxena S., Dally B., Roberts W.L. (2024). Review on production of liquid fuel from plastic wastes through thermal and catalytic degradation. J. Energy Inst..

[50] National Institute of Standard and Technology (2023). NIST 23 Tandem Mass Spectral Libraries.

[51] Auersvald M., Šiman M., Vozka P., Straka P. (2025). Quantitative determination of olefins in pyrolysis oils from waste plastics and tires using selective adsorption by Ag–SiO_2_ followed by GC×GC-FID. Talanta.

[52] Strien J.R.J., Genuino H.C., van Eijk M.C.P., Deuss P.J., Heeres H.J. (2024). Pyrolysis of Polyolefin-Enriched Mixed Plastic Waste Streams: Effects of Pretreatments and Presence of Hydrogen during Pyrolysis. Energy Fuels.

[53] Núñez S.S., Moltó J., Conesa J.A., Fullana A. (2022). Heavy metals, PAHs and POPs in recycled polyethylene samples of agricultural, post-commercial, post-industrial and post-consumer origin. Waste Manag..

[54] Kato L.S., Conte C.A. (2021). Safety of plastic food packaging: The challenges about non-intentionally added substances (NIAS) discovery, identification and risk assessment. Polymers.

