# Supplementary material for: Advanced Analytical Framework for Pyrolysis Product Characterization and Emission Profiling in Mixed Plastic Waste: Implications for Recycling Strategy
Source: Polymers (Basel). 2026 Jun 2;18(11):1381. doi: 10.3390/polym18111381 (PMC13259407; doi:10.3390/polym18111381)
Supplement: Supplementary file 1 [file polymers-18-01381-s001.zip › polymers-4340757-supplementary.pdf]

polymers

MDPI

Supplementary Material

Advanced Analytical Framework for Pyrolysis Product Characterization and Emission Profiling in Mixed Plastic Waste: Implications for Recycling Strategy

Aiping Chen [1,†], Saumitra Saxena [1,*], Vasileios G. Samaras [2] and Bassam Dally [1]

[1] Clean Energy Research Platform, Physical Science and Engineering Division (PSE), King Abdullah University of Science and Technology, Thuwal 23955-6900, Saudi Arabia; aiping@mail.ust.edu.cn (A.C.); bassam.dally@kaust.edu.sa (B.D.)
[2] Analytical Chemistry Core Lab, King Abdullah University of Science and Technology, Thuwal 23955-6900, Saudi Arabia; vasileios.samaras@kaust.edu.sa
* Correspondence: saumitra.saxena@kaust.edu.sa
† Current address: School of Materials Science and Engineering, Suzhou University of Science and Technology, Suzhou 215009, China.

Screening runs at 450 °C: observations from the GC×GC maps

At 450 °C, the polyolefin feeds do not undergo appreciable thermal conversion (mass change <1% by micro-balance; see Table 2 in the main paper). These runs are therefore included solely to document onset behavior and early chromatographic patterning rather than to represent fully developed pyrolysis oils.

This interpretation is consistent with the thermal baseline reproduced in Figure S8, where the principal mass-loss/DTG maxima for the PE/PP-rich samples occur near 460–480 °C. Representative pre-pyrolysis FTIR data for the same sample set are reproduced in Figure S7 to provide the feed context requested during revision. As a practical guide to Figures S1–S6, the lower diagonal band in the contour plots corresponds mainly to linear paraffin/olefin homologues, whereas more displaced clusters indicate branched/cyclic products and the short-^{2}D-retention oxygenate ridge marks oxidized fragments.

Sample-specific notes (P1–P6, 450 °C)

- **P1 (LDPE/HDPE blend).** Partial volatilization dominated by long-chain **n-paraffins** and **α-olefins**; almost no cyclics/aromatics; no discernible oxygenates. The arc peaks at higher carbon numbers and is compressed in the 2D plane relative to 650 °C.
- **P2 (oxidized HDPE + $CaCO_3$).** Hydrocarbon arc similar to P1, but **distinct polar spots** appear at short ^2D times (ketones/aldehydes) arising from pre-oxidized segments; these oxygenates later collapse at 650 °C via decarbonylation/decarboxylation.
- **P3 (PP).** Heterogeneous, “spotty” banding of **branched olefins/paraffins** (C_3–C_{14}), with only trace monocyclic aromatics; oxygenates absent. Overall, lighter and more branched than PE.
- **P4 (PP).** Matches P3: early-cracking **iso-olefin/isoparaffin** features with minimal aromatics and no oxygenates; severity insufficient to drive secondary cyclisation.
- **P5 (oxidized HDPE film + $CaCO_3$).** Similar to P2 but with a **heavier** hydrocarbon distribution and the **strongest oxygenate ridge** among the PE films, mirroring the pronounced carbonyl band and filler seen in bulk characterization.

- **P6 (PE/PP blend).** Composite signature: linear PE-type **n-paraffin/α-olefin** series overlain with PP-type **branched** spots; aromatics and oxygenates remain negligible at this temperature.

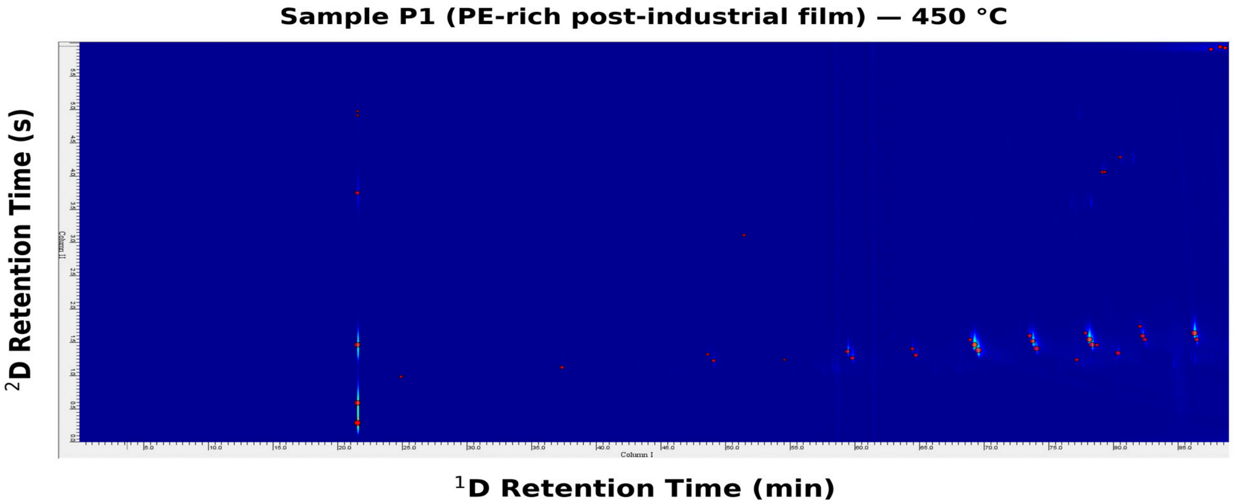

Figure S1: P1 (LDPE/HDPE blend) at 450 °C (GC×GC–TOF–MS 2D contour). ¹D retention time: 0–90 min; ²D retention time: 0–6.0 s. The chromatogram is dominated by a diagonal wax arc of long-chain n-paraffins and α-olefins produced by initial β-scission of polyethylene. Only trace oxygenates are observed.

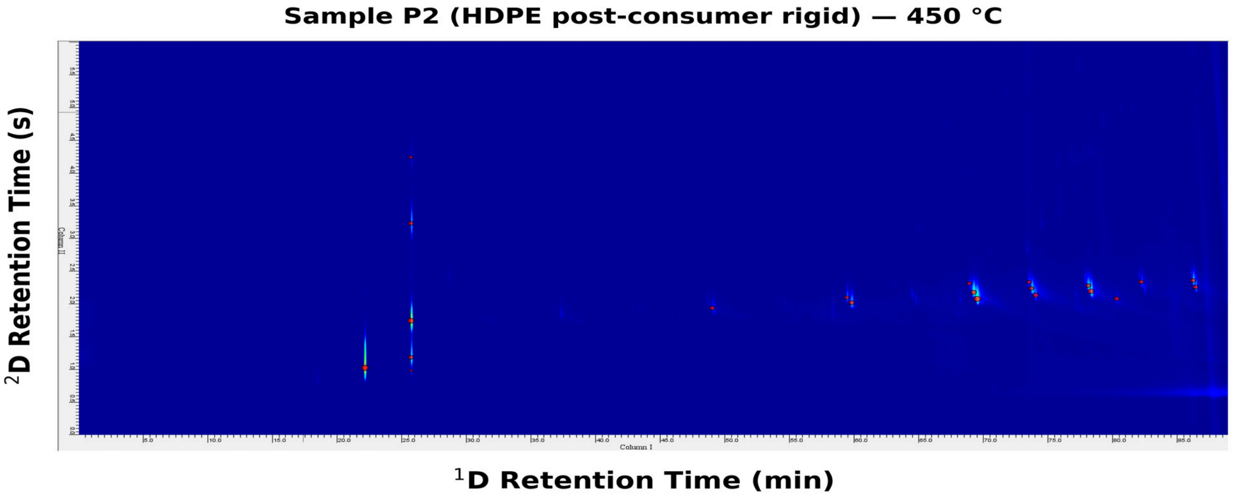

Figure S2: P2 (oxidized HDPE containing ≈6 wt% $CaCO_3$ filler) at 450 °C. ¹D retention time: 0–90 min; ²D retention time: 0–6.0 s. Hydrocarbon features mirror PE, but a polar "oxygenate ridge" (short ²D retention) appears, attributable to ketones/aldehydes from oxidized HDPE. The map records onset behavior rather than full pyrolysis.

https://doi.org/10.3390/polym18111381

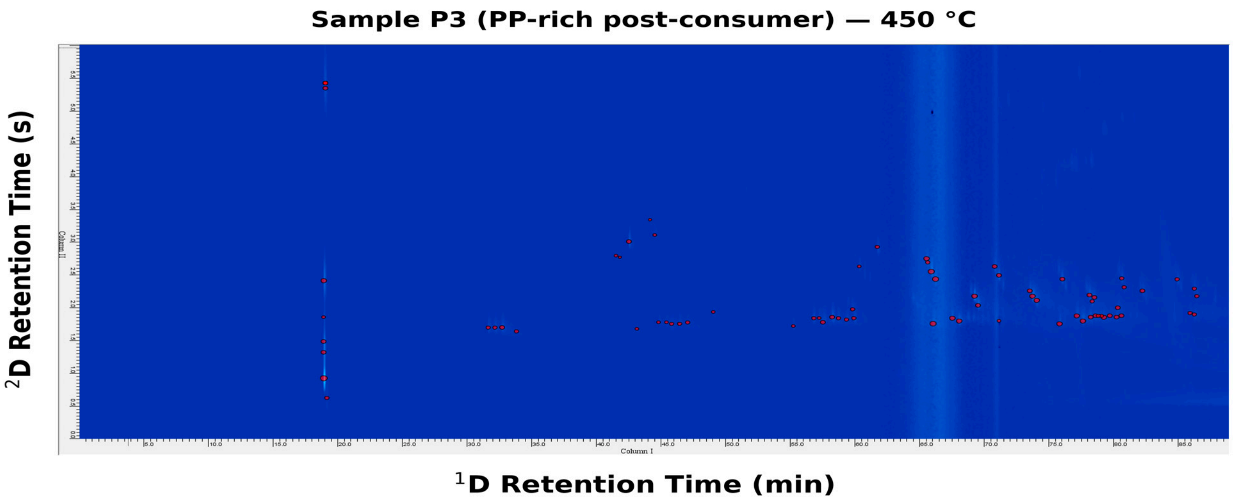

Figure S3: P3 (polypropylene) at 450 °C. ¹D retention time: 0–90 min; ²D retention time: 0–6.0 s. Early-severity PP fingerprint: a spotty envelope of branched olefins/paraffins spanning C_3–C_{14}; aromatics are negligible at this temperature and oxygenates absent. Compared with 650 °C, the products remain heavier and less cyclized.

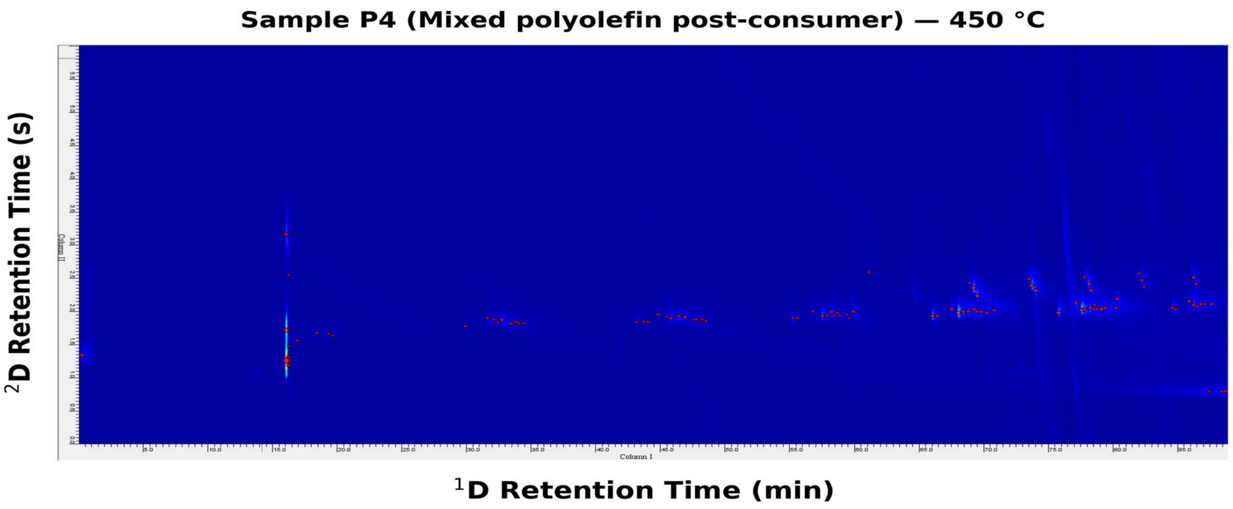

Figure S4: P4 (polypropylene) at 450 °C. ¹D retention time: 0–90 min; ²D retention time: 0–6.0 s. Similar to S3, showing isoparaffin/iso-olefin dominance with minimal aromatization. The map records incipient depolymerization rather than full cracking.

https://doi.org/10.3390/polym18111381

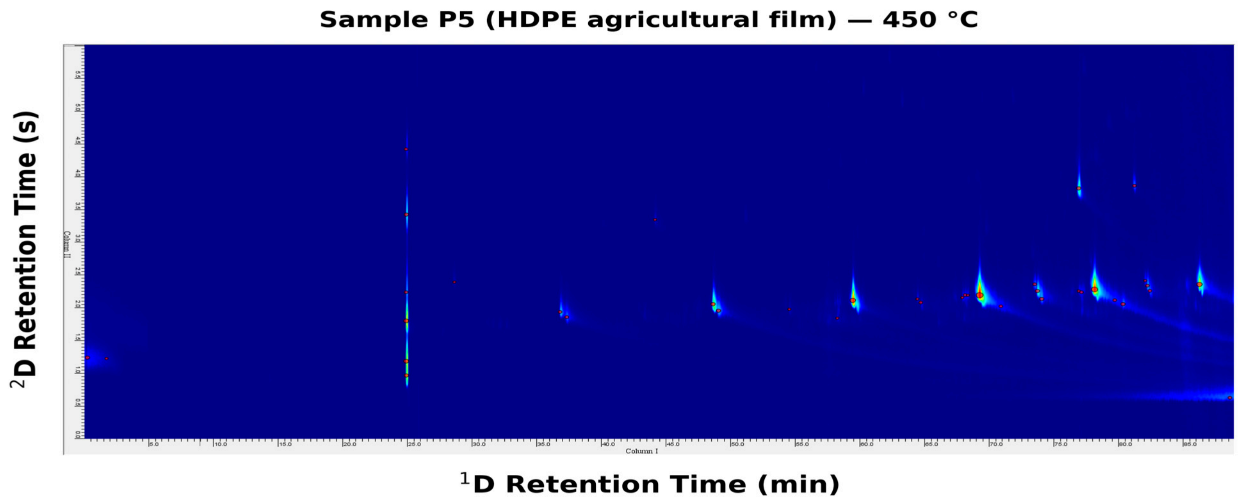

Figure S5: P5 (oxidized HDPE film with ≈10 wt% $CaCO_3$ filler) at 450 °C. ^{1}D retention time: 0–90 min; ^{2}D retention time: 0–6.0 s. PE-type hydrocarbon arc with a prominent oxygenate band, strongest among the films, reflecting weathering prior to analysis. At higher severity (650 °C), these oxygenates diminish but do not fully disappear.

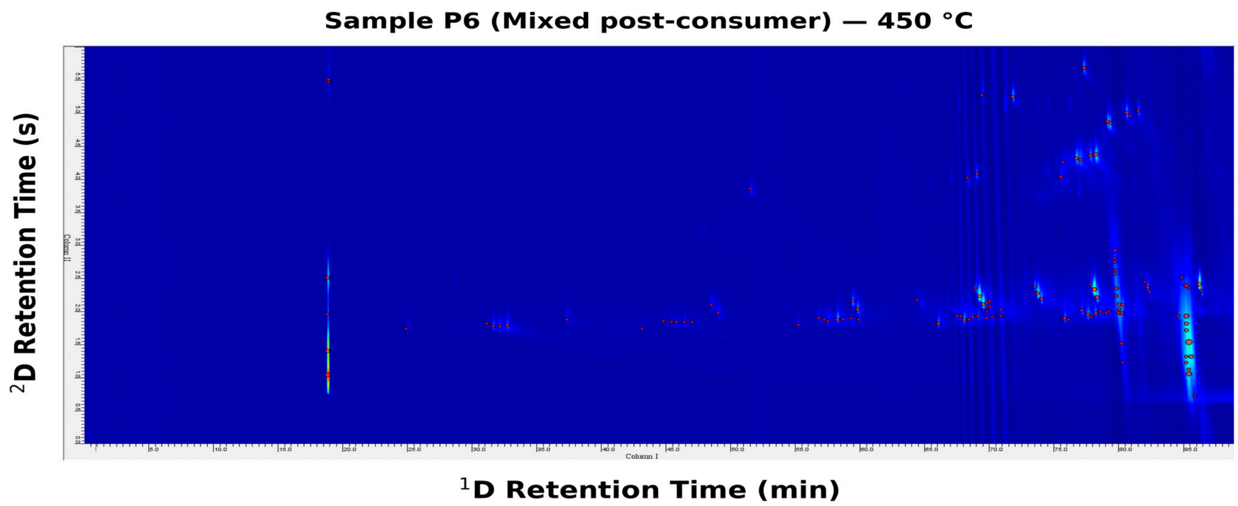

Figure S6: P6 (mixed polyolefin blend: LDPE + HDPE + PP) at 450 °C. ^{1}D retention time: 0–90 min; ^{2}D retention time: 0–6.0 s. Hybrid signature combining linear PE-derived series and PP-derived branched features; aromatics and oxygenates remain near background. Serves as the sub-pyrolytic counterpart to the optimized 650 °C chromatogram in the main text.

https://doi.org/10.3390/polym18111381

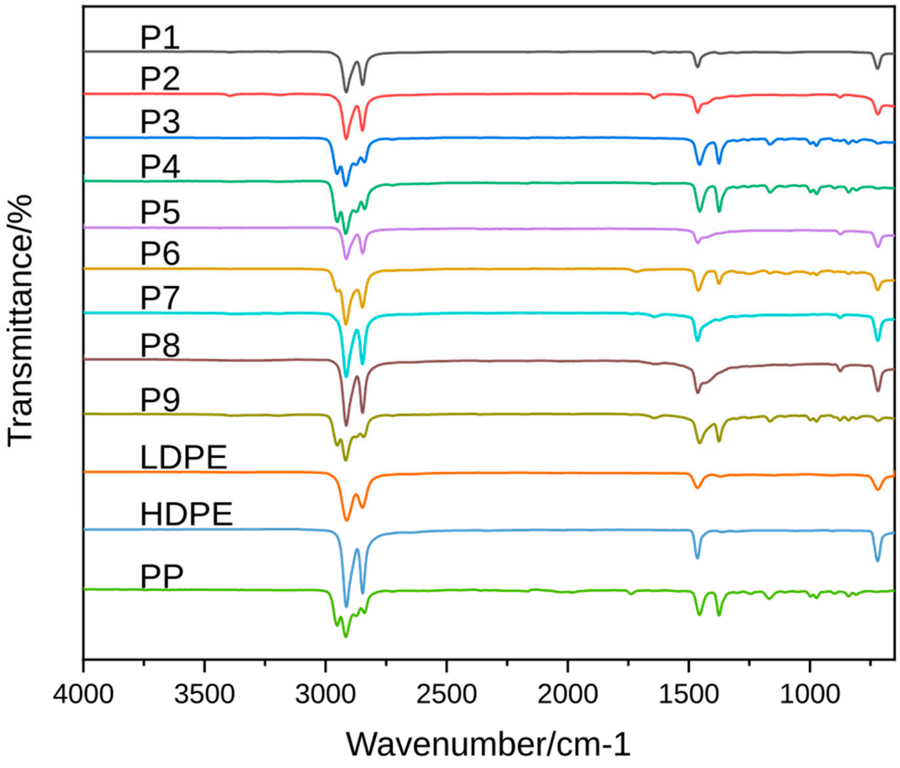

Figure S7: Representative pre-pyrolysis FTIR spectra of samples P1–P9 overlaid with LDPE, HDPE, and PP reference spectra. These data provide the feed-context requested during revision and support the oxidation/polymer assignments used to interpret the chromatographic results.

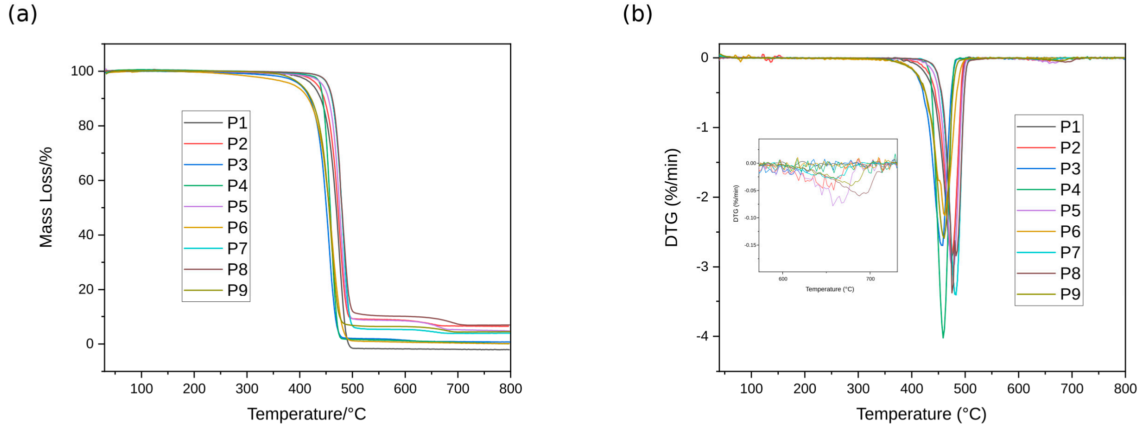

Figure S8: TGA and DTG profiles of samples P1–P9. The principal degradation maxima near 460–480 °C justify the use of 450 °C as a sub-pyrolytic screening condition and 650 °C as the optimized analytical pyrolysis temperature.

Note: Post-pyrolysis residue FTIR was not available in the current dataset; the revision, therefore, includes the pre-pyrolysis FTIR and thermal baseline used to interpret the 450 °C versus 650 °C comparison.

Disclaimer/Publisher's Note: The statements, opinions and data contained in all publications are solely those of the individual author(s) and contributor(s) and not of MDPI and/or the editor(s). MDPI and/or the editor(s) disclaim responsibility for any injury to people or property resulting from any ideas, methods, instructions or products referred to in the content.
